# Climate refugia in the Great Barrier Reef may endure into the future

**DOI:** 10.1126/sciadv.ado6884

**Published:** 2024-11-29

**Authors:** Chaojiao Sun, Craig Steinberg, Eduardo Klein Salas, Camille Mellin, Russell C. Babcock, Andreas Schiller, Neal E. Cantin, Jessica S. Stella, Mark E. Baird, Scott A. Condie, Alistair J. Hobday, Mike Herzfeld, Nicole L. Jones, Xuebin Zhang, Matthew A. Chamberlain, Russ Fiedler, Cody Green, Andrew D. L. Steven

**Affiliations:** ^1^ CSIRO Environment, Perth, Western Australia, Australia.; ^2^ Australian Institute of Marine Science, Townsville, Queensland, Australia.; ^3^ Integrated Marine Observing System, University of Tasmania, Hobart, Tasmania, Australia.; ^4^ University of Adelaide, Adelaide, South Australia, Australia.; ^5^ CSIRO Environment, St Lucia, Queensland, Australia.; ^6^ CSIRO Environment, Hobart, Tasmania, Australia.; ^7^ Institute for Marine and Antarctic Studies, University of Tasmania, Hobart, Australia.; ^8^ Great Barrier Reef Marine Park Authority, Townsville, Queensland, Australia.; ^9^ University of Western Australia, Perth, Western Australia, Australia.

## Abstract

Although global warming is leading to more frequent mass coral bleaching events worldwide, parts of the Great Barrier Reef (GBR) have consistently escaped severe coral bleaching. Modeling and satellite observations show that climate refugia are created by the upwelling of cooler water to the surface through the interactions of tides and currents with dense reef structures. Here, we use a high-resolution nested regional ocean model to investigate the future status of two relatively large refugia. On the basis of model projections under a high-emission scenario, we find that the upwelling mechanisms will stay active in a warming climate, and these regions are likely to remain approximately more than 1°C cooler than surrounding waters until at least into the 2080s, providing thermal relief to corals. Identification and protection of these refugia may help facilitate reef survival and related biodiversity preservation by allowing their corals time to acclimatize and adapt and ultimately provide source populations to replenish the rest of the reef.

## Teaser

High-resolution regional ocean model suggests that climate refugia in the Great Barrier Reef can persist at least into the 2080s.

## INTRODUCTION

Coral reefs around the world face numerous challenges such as storm damage, predation, pollution, disease outbreaks, overfishing, and climate change. Among these, climate change is the greatest threat to coral reefs (*1*, *2*), which are highly sensitive ecosystems vulnerable to changes in temperature, ocean chemistry, and other environmental factors. Coral bleaching occurs when corals experience prolonged heat stress and expel the symbiotic algae living in their tissues, causing the corals to turn white (bleach). Corals can recover from bleaching if the thermal stress is not too severe or prolonged; otherwise, they are susceptible to mortality.

As ocean temperatures rise, mass coral bleaching events have increased, in terms of frequency, spatial reef area affected, and the intensity of heat stress, a trend that has been predicted (*1*) and observed worldwide (*2*). Climate change can also exacerbate other impacts on corals from sea level rise, changes in storm patterns, altered ocean circulation patterns (affecting food availability and larval dispersal), to the crown-of-thorns starfish (COTS) predation (*2*–*6*). In addition, increased carbon dioxide emissions directly contribute to ocean acidification, which affects coral growth and structural integrity (*7*).

The Great Barrier Reef (GBR) is the world’s largest coral reef ecosystem, with over 3000 individual reefs inside the GBR Marine Park and an additional 680 reefs situated in the adjoining Torres Strait region. About 99% of the UNESCO World Heritage Area is within the GBR Marine Park, which spans over 2300 km of coastline and encompasses an area of 344,400 km^2^ (*8*). The average global ocean surface temperature has increased by 0.88°C from 1850 to 1900 to 2011 to 2020 (*9*), with the GBR warming by 0.8°C in the same period (*10*, *11*). This trend is projected to continue throughout the 21st century (*12*).

Even during the most extensive global bleaching events, some regions have consistently escaped severe coral bleaching, suggesting that they could be climate refugia (*13*–*15*). Climate refugia for coral reefs refer to specific geographic locations that retain favorable environmental conditions for corals, even when the surrounding areas experience inhospitably elevated temperatures due to the effects of climate change (*16*). Mesoscale eddies can also affect warming patterns, but local-scale patterns are usually not resolved by climate models. These local processes, such as upwelling induced by wind, currents and tides, can create and maintain thermal refugia by moving cooler water from depth toward the surface (upwelling) (*17*–*20*); however, these processes are usually not resolved by climate models, which have a typical resolution around 100 km. Furthermore, climate models do not resolve fine-scale topography associated with small islands and coral atolls, which can mitigate the surface warming by interacting with currents and tides to facilitate upwelling and enhance vertical mixing. For example, enhanced upwelling associated with future strengthening of the equatorial undercurrent is found to mitigate the surface warming around several equatorial Pacific islands and atolls providing crucial refuges for marine biodiversity, but climate models did not resolve these topographic features and failed to identify such refugia (*15*).

To evaluate the vulnerability of climate refugia to global warming, high-resolution modeling that can better resolve local dynamics is needed. Several studies applied various statistical and semidynamic downscaling methods, but the fate of climate refugia in the GBR remains uncertain as very different outcomes are projected by these studies (*6*, *20*–*22*). Dynamical downscaling involves driving a higher spatial resolution regional ocean model with climate model output or reanalysis data to produce a detailed simulation of local climate conditions using explicit representations of important physical processes. However, dynamic downscaling is computationally expensive and often considered prohibitive (*6*, *23*, *24*). To date, no study has investigated future conditions in the GBR using fully three-dimensional ocean general circulation models with a horizontal resolution finer than 10 km.

The aim of this study is to identify potential climate refugia in the GBR and assess whether these refugia will persist, with the climate refugia defined as areas where the sea surface temperature (SST) is more than 1°C cooler than surrounding waters. We first identify thermal refugia within the GBR through high-resolution regional ocean modeling at 4-km resolution. We then assess the long-term fate of these refugia using dynamical downscaling using the same model, which resolves local oceanographic processes. The regional model for the GBR is nested within a near-global model at 10-km resolution, which simulates large-scale ocean circulation and provides the initial condition and boundary conditions for the regional model. Both models are driven by meteorological conditions derived from a high-emission scenario, the Representative Concentration Pathway 8.5 (RCP8.5) (*25*). Although the RCP8.5 emission scenario is now considered a lower probability scenario, refugia existing under RCP8.5 are very likely to persist under any realistic climate future. The output from the regional model is then used to assess the fate of identified climate refugia in the GBR. We show that the climate refugia regions in the GBR are likely to remain about 1°C cooler than surrounding waters at least into the 2080s. If realized, then these regions are less likely to experience temperatures that exceed regional thermal limits of corals due to their high regional connectivity.

## RESULTS

### Present-day climate refugia identified from model and satellite observations

**Fig. 1.**
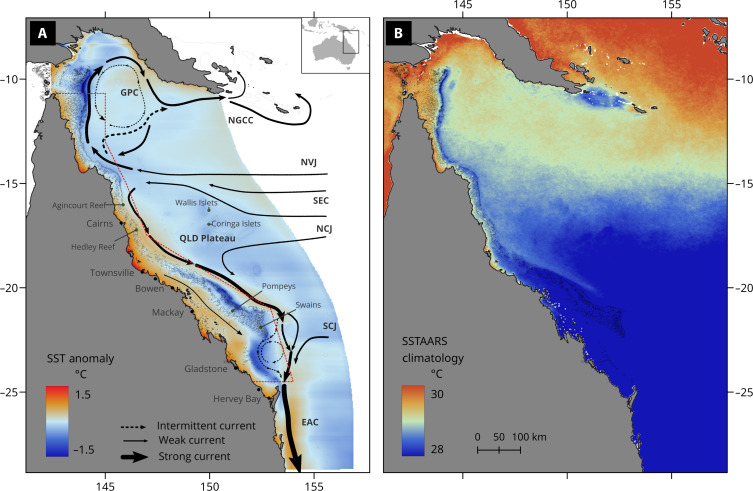
Schematic of Coral Sea circulation indicating regions of upwelling and SST anomaly during austral summer (January to March) and satellite observations of SST over the same season. (**A**) Major currents in the Coral Sea (arrows) and mean SST anomalies averaged over the austral summer (color shadings). The size of the arrow is indicative of the strength of the current. Color shadings indicate mean SST anomalies calculated from eReefs 4-km model (GBR4) simulations (*41*) averaged over the summer months from 2010 to 2020. Negative values (blue) denote regions with temperatures cooler than surrounding waters along the same latitude, while positive values (red) denote temperatures warmer than surrounding waters along the same longitude. The major ocean currents are denoted by various arrows and acronyms: Southern Equatorial Current, SEC; North Vanuatu Jet, NVJ; North Caledonian Jet, NCJ; South Caledonian Jet, SCJ; East Australian Current, EAC; Gulf of Papua Current, GPC; and New Guinea Coastal Current, NGCC. The red dashed line denotes the boundary of the GBR Marine Park. (**B**) Satellite SST observations showing persistent upwelling in the austral summer season (January to March). High-resolution (2 km) SST climatology averaged over the summer season is calculated from the SSTAARS.

Two large GBR thermal refugia are identified using a high-resolution regional ocean model that resolve tides and fine-scale topography (see Materials and Methods for details). One refugium is in the Southern GBR at the outer shelf edge (off the Whitsundays, Pompeys to the Swains reef complexes), while the other is in the Northern GBR along the outer shelf from the eastern Torres Strait to the ribbon reefs off Cape York (Fig. 1).

We define refugia as areas where the SST is more than 1°C cooler than the surrounding waters during the warm season (January to March), using hourly SST data from our regional model. We calculate hourly SST anomalies at each location by removing the longitudinal averages along a transect from the coast to the open ocean. We first average SST values at each latitude at each hour along a longitudinal transect—from the coast to 200 km past the edge of the continental shelf (defined by the 200-m isobath). Next, we compare the SST at each grid point along the transect with this average to calculate an anomaly value. The SST anomaly $T^{\prime }$ is thus calculated as

$$T^{\prime }=T-\bar{T}$$ (1)

Here, T is the SST at each grid point, and $\bar{T}$ is the SST averaged over the longitudinal transect.

Refugia are the upwelling locations where the hourly anomaly SST values, averaged over the warm season, are lower than −1°C. This approach of identifying upwelling locations using SST anomalies over a longitudinal transect from the coast to open ocean at each latitude has been used previously to identify cool SST fronts with satellite observations (*26*). The 1°C threshold is also the basis for the common definition of cumulative heat stress used in the context of coral bleaching such as degree heating week (DHW), which is based on the number of days with SST more than 1°C above the maximum monthly mean (MMM) over the previous 3 months, e.g., (*27*). Because the shelf break is oriented mostly along the north-south direction in the GBR, we use the temperature anomalies relative to the same latitude to highlight the role of shelf break and outer reef topography on the impact of surface temperatures due to tidal pumping. By removing the mean temperature along a longitudinal transect from the coast out to the open ocean at 200 km past the continental shelf break, we capture the areas that are cooler than in both inshore and offshore waters.

High-resolution daily satellite SST observations show that a cooler SST persists along the GBR continental shelf throughout the summer (Fig. 1B). The high-resolution (~2 km) satellite SST daily climatology, the Sea Surface Temperature Atlas of the Australian Regional Seas (SSTAARS), was constructed using nighttime only and nearly cloud-free data from 1992 to 2016 to reduce diurnal bias and cloud contamination (*28*). The same summertime SST climatology has also been calculated with the National Oceanic and Atmospheric Administration (NOAA) Coral Reef Watch (CRW) 5-km data (*27*, *29*). Similar patterns are seen, but the narrow band of upwelling regions becomes more diffuse due to the dataset’s coarser resolution (f. S1). More information about the SSTAARS and NOAA SST data is provided in Materials and Methods.

**Fig. 2.**
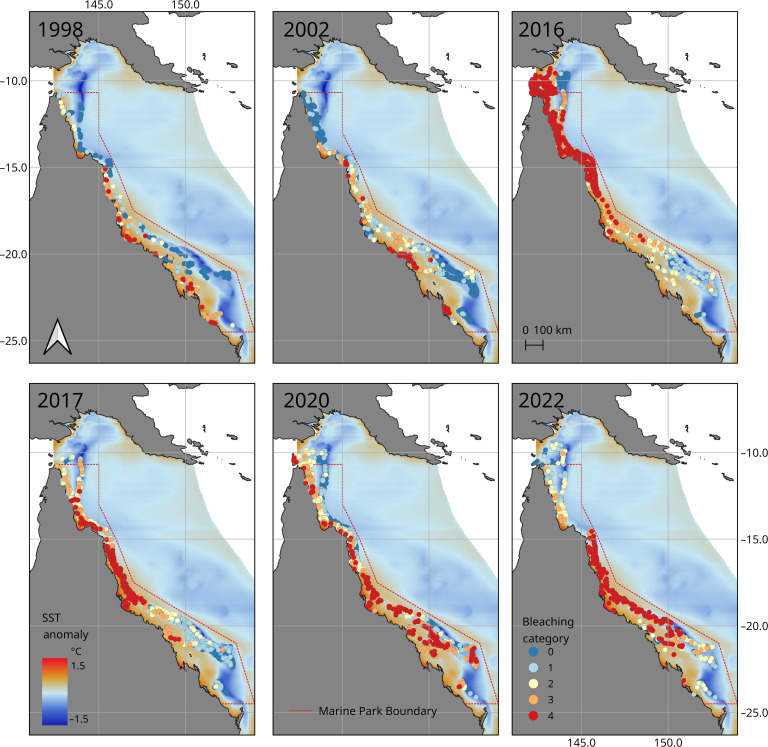
Spatial extent and reef-level prevalence of shallow-water (<6 m) coral bleaching during six consecutive mass bleaching events on the GBR. The 10-year climatology of surface temperature anomaly from the GBR4 model is represented by color shadings (same as in Fig. 1A). Aerial survey bleaching scores for the bleaching events in 1998, 2002, 2016, 2017, 2020, and 2022 are as follows: blue, <1% of shallow-water corals bleached; light blue, 1 to 10%; yellow, 10 to 30%; orange, 30 to 60%; and red, >60%. The number of reefs surveyed each year was 587 in 1998, 630 in 2002, 1135 in 2016, 742 in 2017, 1036 in 2020, and 719 in 2022 (*30*).

These two refugia areas identified by the model are broadly consistent with the regions that have consistently escaped severe coral bleaching during recent coral bleaching events from 1998 to 2022 as determined by aerial surveys (Fig. 2) (*2*, *30*–*33*). The median continuous estimate of the percentage of coral cover bleached from the in-water surveys displays a strong relationship and agreement to the categorical aerial bleaching score during the 2016 bleaching event on the GBR [e.g., Supplementary Information in (*2*)]. Because aerial bleaching provides the highest spatial coverage over the GBR, and the two approaches capture similar estimates of bleaching prevalence, [e.g., see the Extended Data Figure 5 in (*2*)], the aerial bleaching observation data are the most spatially robust dataset available for comparison with large spatial oceanographic drivers of thermal stress. Using the NOAA CRW 5-km-resolution SST data (*29*), qualitative comparison of the temperature anomalies for each bleaching event with the aerial surveys showed a general agreement in the northern refuge but the southern refuge is not obvious (fig. S2).

### Mechanisms of upwelling in the refugia

The general agreement among ocean circulation models, satellite observations, and aerial surveys suggests that these refugia exist because of enduring local physical oceanographic processes or meteorological forcing in the summer. A number of mechanisms provide regular upwelling events to create effective refugia along the outer reef and shelf break, such as complex topography (the continental slope and reef structures) interacting with the boundary and tidal currents (*17*, *34*, *35*), upwelling favorable winds (*36*, *37*), cyclonic eddies (*38*, *39*), and internal waves (*40*). Upwelling is prevalent during January to April in the GBR when the southeast trade winds are weak and moderate northwest monsoon winds prevail (*41*).

**Fig. 3.**
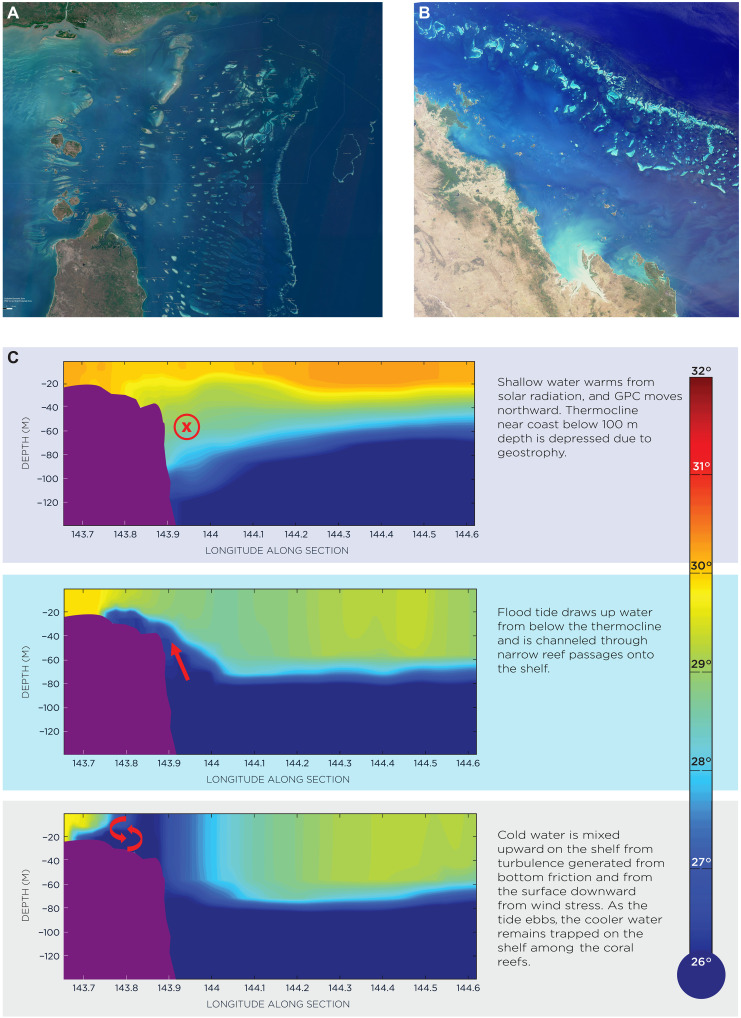
Schematic representation of the tidal-driven upwelling over a tidal cycle in the far northern GBR region and satellite images showing densely packed reefs in the Northern and Southern GBR. (**A**) Satellite image showing the densely packed reef structures in the Northern GBR and Torres Strait [Credit: E. Lawrey (2013); Torres Strait Clear sky Landsat (eAtlas, Australian Institute of Marine Science); source: NASA; https://eatlas.org.au/data/uuid/71c8380e-4cdc-4544-98b6-8a5c328930ad]. (**B**) Image of the Southern GBR Pompeys region (courtesy of NASA/GSFC/LaRC/JPL, MISR Team); https://earthobservatory.nasa.gov/images/1337/australia146s-great-barrier-reef. (**C**) Schematic representation of the tidal-driven upwelling over a tidal cycle in the far Northern GBR region over a depth-longitude cross section. The red symbol ⊗ denotes the direction of the northward flowing GPC (flowing away from the reader perpendicularly into the paper).

When comparing the Ocean Forecasting Australia Model (OFAM) and GBR4 model performance, including tides in the GBR4 model shows that the upwelling is more accurately resolved compared with the Australian Integrated Marine Observing System (IMOS) high-resolution (~2 km) SST data (see fig. S4 in the Supplementary Materials) (*42*). The upwelling observed in the northern refugium (in the eastern Torres Strait and off Cape York in the Northern GBR) is mainly due to vigorous tidal mixing in the region and the presence of dense reef structures with narrowly spaced reef channels facilitating onshore and upward flow of cool water (*28*). A schematic of such a scenario is shown in Fig. 3. Despite the fact that the northward flowing Gulf of Papua Current (GPC) suppresses upwelling by lowering the thermocline (fig. S3), the presence of a sloping shelf break diverts the current away from the outer reefs and creates a surface divergence that enables upwelling to occur along the fringing reefs (*41*). The densely packed meandering outer reefs facilitates the formation of anticyclonic eddies in the embayments. These eddies further contribute to the favorable conditions for upwelling in the region.

The upwelling in the southern refugium (in the Swains/Pompeys region) is mainly caused by strong tidal mixing (*28*) and the influence of the East Australian Current (EAC) (*38*). The geostrophic balance between the Coriolis force and horizontal water pressure gradient causes the southward flowing EAC to lift the thermocline along the continental shelf break (fig. S3). This allows cooler water below the mixed layer to flow onto the shelf, where it is mixed upward by strong tidal currents flowing through narrow channels in the presence of dense reef structures (“tidal pumping”) (Fig. 3B) (*43*).

### Large-scale warming patterns and connectivity

The GBR marine environment is strongly influenced by the large-scale circulation in the Coral Sea (*44*, *45*). Changes in the western boundary currents are expected to influence the thermal structure of the reef and affect larval dispersal and connectivity within the GBR (*20*, *46*). Although climate models (horizontal resolution of ~100 km) could simulate changes in the volume transport of western boundary currents reasonably well as they are driven by basin-scale winds, local details of these currents such as their widths and maximum speeds are often poorly simulated (*47*). Here, we reveal large-scale changes around the GBR between the present-day climate and future climate at the end of this century using a 10-km-resolution near-global ocean model, the OFAM version 3 (OFAM3) (*48*), forced with climate change signals from 2006 to 2100 from an ensemble average of 17 climate models under the high-emission scenario RCP8.5 (see Material and Methods for details) (*49*, *50*).

**Fig. 4.**
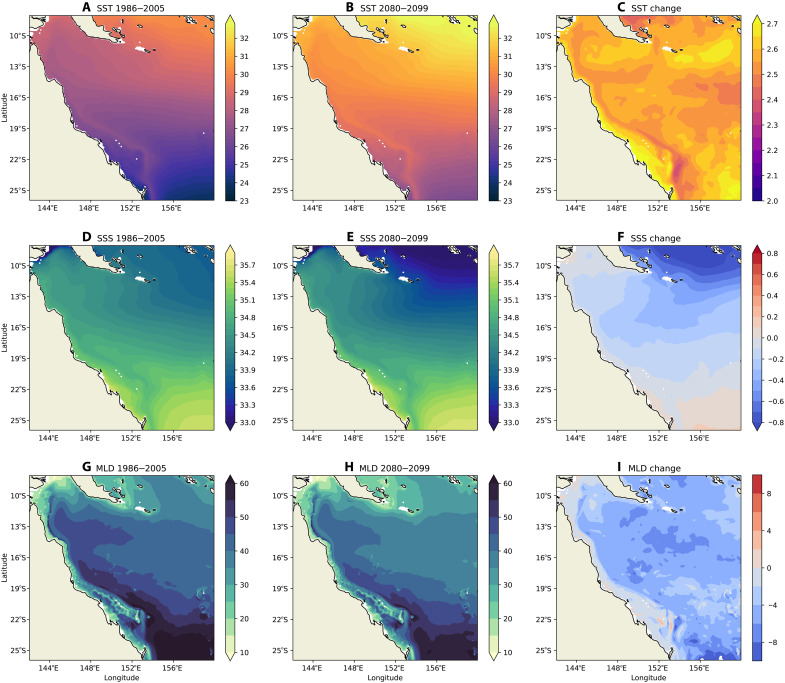
Large-scale patterns and changes in SST, SSS, and mixed layer depth. The data are presented for two 20-year periods in the present-day climate (1986 to 2005) and future climate (2080 to 2099). (**A** to **C**) SST. (**D** to **F**) SSS. (**G** to **I**) Mixed layer depth (MLD).

The OFAM3 model does not simulate tides explicitly and lacks the horizontal resolution to resolve reef features at scales less than 10 km; therefore, it does not capture the refugia areas identified by the regional model GBR4 (fig. S4). However, the OFAM3 model offers valuable insight into ocean warming and potential changes to ecological connectivity under climate change. The surface waters in the GBR are projected to warm by about 2.7°C by the end of the 21st century (Fig. 4, A to C), which will likely cause mass coral bleaching all over the reef (*22*). The sea surface salinity (SSS) will become fresher in the open ocean (Fig. 4, D to F), consistent with the trend over the past 60 years (*35*). The average surface mixed layer depth, defined as the depth over which the potential density increases by 0.3 kg m^−3^ from the surface value (*48*), decreases by around 5 m or 10% off the shelf (Fig. 4, G to I). Because of the warming and freshening, the density of the mixed layer becomes lighter and the density gradient between the mixed layer and deeper water likely becomes higher, making the surface mixed layer more stable. On the other hand, a shallower mixed layer could make it easier to entrain deep water under conditions such as vigorous tidal mixing.

The broad westward South Equatorial Current (SEC) bifurcates into two energetic western boundary currents upon reaching the Australian continental shelf: the equatorward GPC and poleward EAC (Fig. 1). The location of the SEC bifurcation separates the warm tropical gyre in the north and cool subtropical gyre in the south. The bifurcation location varies seasonally and annually, as well as with depth, influenced by the vertical current structure in the region (*44*, *51*), with an annual average between about 14.4°S and 14.7°S (*52*). Future changes in the SEC bifurcation latitude are crucial for understanding the ecological implications of climate-driven shifts in ecosystem connectivity and designing protection and intervention strategies by management agencies (*22*). Any shift in the bifurcation will likely affect connectivity for reefs located between the current and future locations of the bifurcation and affect coral recruitment through long-distance dispersal events that are critical to coral recovery on isolated reefs (*20*).

**Fig. 5.**
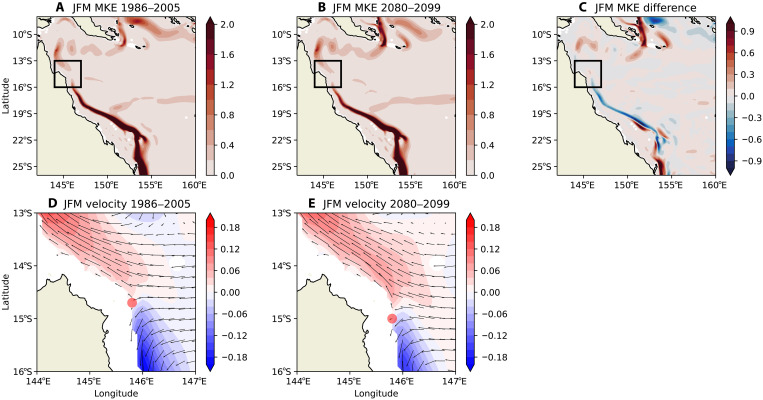
Changes in strengths of ocean currents and SEC bifurcation location in the future for the warm season January-February-March. Mean kinetic energy (MKE) climatology (over 20 years) (in meters squared per seconds squared) integrated over the top 50 m in the (**A**) present-day climate (1986 to 2005) and (**B**) future climate (2080 to 2099). (**C**) Difference in MKE between the future and present-day climate. (**D** and **E**) Mean surface velocity (arrows) and meridional surface velocity (in meters per second) (colors). Note that the SEC bifurcation latitude in the present and future climate is marked by a red dot in (D) and (E). The extent of the plot in (D) and (E) is denoted by the rectangles in (A) to (C). Arrows in (D) and (E) denote the current direction and speed, while colors show the meridional velocity with the transition from blue to red indicating the SEC bifurcation. JFM, January-February-March.

The OFAM3 dynamically downscaled 100-year ocean projections at 10-km resolution reveal substantial future changes in the large-scale surface currents in the GBR region. The GPC is projected to strengthen, consistent with two generations of climate model simulations (*47*), which will likely bring more cooler water to the north. The strengthening of the GPC is due to increased contributions from the SEC via the North Vanuatu Jet and North Caledonian Jet (Fig. 1A). The nascent EAC portion in the central GBR (between about 16°S and 20°S) is expected to weaken (Fig. 5, A to C).

The SEC bifurcation location shifts southward slightly during the warm season (January to March) in the future (Fig. 5, D and E). The biggest shift of ~1° latitude happens in the austral autumn months of March, April, and May between the two 20-year periods (fig. S5), while the shifts in other seasons are less prominent (figs. S6 to S8). The southward shift is correlated with the southward shift of zero wind stress curl according to the Sverdrup dynamics with the exact location influenced by bathymetric steering and jet formation (fig. S9) (*44*, *53*). The annual average SEC bifurcation latitude moved from the current location of ~14.5°S (*52*) to ~15.7°S toward the end of this century with the bifurcation point moving southward by an average of about 1 km/year (fig. S9). Our finding further extends previous research showing that the SEC bifurcation has been shifting southward over the past 60 years (*51*, *54*).

### Persistence of climate refugia into the future

Here, we use the regional model GBR4 to simulate local conditions for two decades in the future, 2050s and 2080s. This regional model was nested inside the near-global ocean model OFAM3, both driven by the same atmospheric conditions derived from climate model outputs under the high-emission scenario. The OFAM3 model provides the initial condition and boundary conditions for the GBR4 model to account for predicted changes in large-scale circulation and thermal structure in the GBR discussed in the last section.

We use the same tidal constituent amplitude and phase of constituents in the present-day climate for the future simulations; therefore, the influence of sea level rise on tidal dynamics has not been considered here. Several studies have investigated the influence of sea level rise on tidal dynamics in coral reef systems and found that the response to sea level rise is complex, nonlinear, and spatially variable, with tidal range experiences up to 10% variation dependent on specific locations (*55*–*57*). We hypothesize that the impact of sea level rise to the mixing role of the tides on the climate refugia that are located the outer continental shelf of the GBR is minimal.

**Fig. 6.**
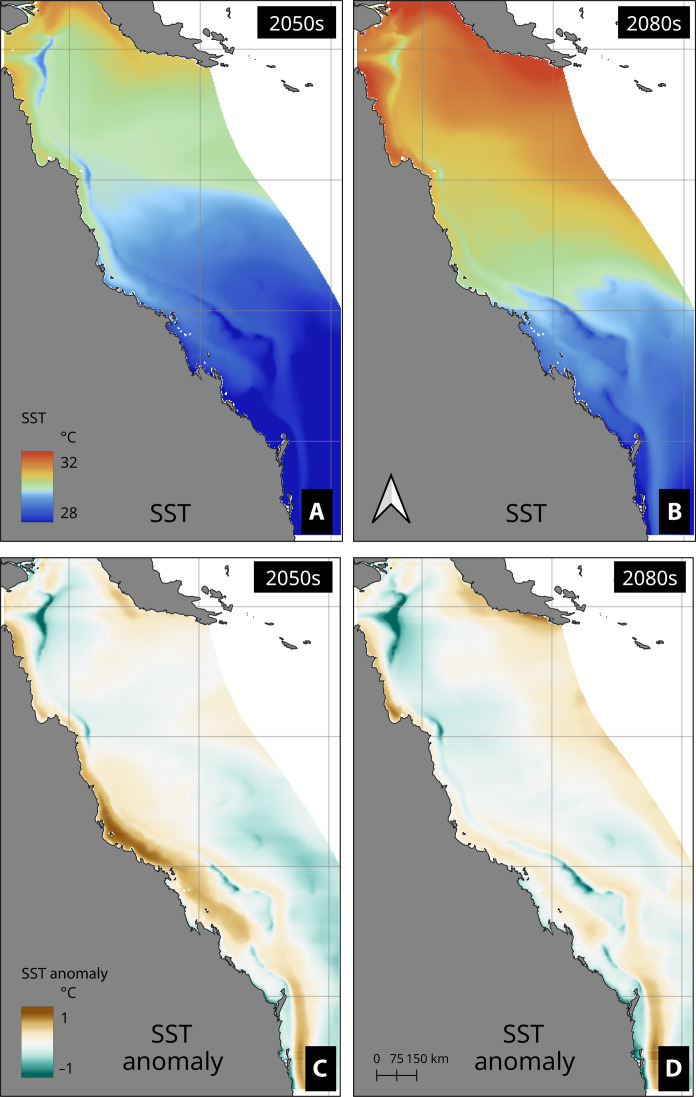
Summertime decadal mean SST and latitudinal SST anomalies showing the warming patterns in the GBR and persistence of upwelling in the refugia areas. Summertime decadal mean SST over (**A**) 2050s and (**B**) 2080s. SST anomalies over (**C**) 2050s and (**D**) 2080s.

There is a notable increase in SSTs in the 2080s compared to those in the 2050s as a consequence of long-term warming (Fig. 6, A and B). However, the cooler temperatures along the continental shelf are clearly visible, driven by the same upwelling processes as those observed in the present day. In both periods, the cooler temperatures (compared to the ambient temperatures) identified in the historical period persist through both decades, constituting climate refugia (Fig. 6, C and D).

## DISCUSSION

Using a regional ocean model that resolves tides and local topography, we have identified two large contemporary climate refugia in the northern and southern GBR, demonstrating the importance of local processes in characterizing climate refugia to inform climate-smart spatial planning to conserve biodiversity (*58*, *59*). Reef-scale oceanographic processes, especially the interaction of complex reef topography with tides and the channeling of currents through dense reef structures, are primarily responsible for localized upwelling events in the refugia. In contrast, in the central GBR, while deeper cold waters intrude onto the continental shelf during austral summer, they usually do not reach the surface and the upwelling intrusion often corresponds to surface warming due to small tidal currents and less dense reef structures (*31*, *37*). Satellite observations (*28*, *29*) and aerial reef surveys of coral bleaching over the past six bleaching events broadly confirm the existence of climate refugia identified by our model.

Using the same regional model nested inside a 10-km-resolution near-global model, we show that climate refugia could persist at least into the 2080s under the high-emission scenario RCP8.5, with surface water in these areas continuing to be more than 1°C cooler than surrounding waters, providing thermal relief to corals in those areas. These results likely represent a worst-case scenario as they are derived under the high-emission scenario RCP8.5, which now looks unlikely to be realized due to global climate actions (*25*). Coral reefs inside climate refugia could potentially provide source populations to replenish the rest of the reef, which should motivate renewed efforts to protect them.

Our metric for defining climate refugia (regions where the summertime SST remains 1°C cooler than surrounding waters) differs from other studies, which usually use a measure of accumulated heat stress, the DHW (*21*, *22*). For example, one study uses the metric of a probability of thermal stress event (DHW > 4°C-weeks) being less than one event every 10 years and finds that 84% of global coral reef pixels in the present-day climate are thermal refugia, but only 0.2% remain at 1.5°C warming and 0% at 2.0°C of global warming (*21*), while another study uses the metric of the lowest 20% of climatological DHWs and find that climate refugia will fail when global warming exceeds ~3°C (*22*).

Our metric is a measure of persistent upwelling, which captures relative bleaching risk rather than the actual bleaching risk. As regional corals are likely to have similar thermal tolerances because of the high degree of larval connectivity, corals inside the refugia will have a similar heat tolerance level as the corals outside the refugia. This same connectivity also ensures that coral populations within the refugia do not become so locally adapted that they are unable to survive outside of them. During a bleaching event, the accumulated heat stress (e.g., DHWs) would be similar for the corals inside and outside of the refugia as the overall warming rate would be the same. However, corals inside the refugia will have a better chance of staying below the bleaching threshold and avoiding severe bleaching, provided that the upwelling is active while the warming event occurs (*60*). During the 2016 and 2020 bleaching events, the cumulative heat stress in the northern refugia area was much higher than normal but corals inside the refugia escaped severe bleaching (*61*).

Our approach to compare temperatures with surrounding waters is similar to the one described in (*62*), which used a shifting baseline to identify future local marine heat waves that are relatively short-lived and intense on top of a long-term warming trend, while the fixed baseline approach identifies total heat exposure of marine organisms, which is relevant for studying impacts on ecosystems (*63*). Our approach may also be more relevant from an ecological perspective. For example, historical conditions may become less relevant if coral populations can acclimatize and adapt to broad-scale temperature trends, which could occur to some degree under strong climate mitigation (*22*). However, local refugia will continue to be critical wherever changes are too rapid or beyond the inherent capacity of corals to adapt. We note that the question of whether coral communities can acclimate or adapt rapidly enough in these upwelling areas needs to be addressed by further research.

In this study, we have not focused on the detailed biological response to predicted warming patterns, such as water depth dependence. Nevertheless, the close correspondence between the cooler upwelling regions and rarely bleached coral reefs in aerial surveys is so compelling that future protection of long-term climate refugia, such as those identified here, would likely increase the overall resilience of the GBR.

The shift in bifurcation and the weakening of the EAC in the central GBR may reduce the size of the region contributing heat-adapted coral larvae to the central and southern GBR, as well as slowing their movement, although such effects are expected to be limited by the small extent of the shift during November/December when most corals on the GBR spawn. It is not yet known whether a warming climate will change the timing of spawning of corals. A recent study has found that the onset of coral bleaching will shift from late summer to spring in most coral reef regions around the world due to projected longer periods of heat stress in the future (*64*). The region of the GBR at which the SEC bifurcation occurs is also ecologically important for the COTS, a coral predator that is a major cause of coral loss on the GBR (*65*). It is unclear what the impact of bifurcation change will be on COTS outbreak. Nevertheless, it is important to highlight the potential for future changes in latitude and timing in the SEC bifurcation for the ecology of reef.

Our study emphasizes the critical significance of safeguarding climate refugia areas against various threats, such as pollution, outbreaks of coral-eating COTS, and overfishing, so as to maximize the potential benefits of these refugia (*66*). Although the extent of natural acclimation and adaptation capabilities of corals is not yet fully understood, they have the potential to play a crucial role in the long-term survival of coral reefs (*67*). Over time, the ability of corals and their symbiotic algae to develop heat-tolerant adaptations can contribute to maintaining the integrity of the refugia as the refugia areas will continue to warm under climate change despite their natural resiliency due to local oceanographic conditions. Consequently, preserving these refugia becomes essential for the long-term viability and resilience of coral reef ecosystems.

It is crucial that major reef restoration initiatives prioritize these refugia as they are likely to continue to be the most favorable locations for sustained coral growth and health. The protection of these potentially high-value refugia sites offers the best opportunity for natural adaptation and effective interventions to ensure the future security of the reef. Both southern and northern refugia will need to be fully protected. For example, although some reefs within the refugia are located inside the GBR Marine Park, but not all of them are fully protected, 17% of the refugia are for general use, 20% are for habitat protection, and 63% are classified as Marine National Park (see fig. S10). A substantial portion of the refugia in the eastern Torres Strait is outside the GBR Marine Park boundary and managed by the Torres Strait Regional Authority. We recommend that these high-value climate refugia be designated as high-priority protected areas to maximize success for natural adaptation and potential deployment of large-scale human interventions, such as assisted colonization, assisted evolution, or cloud brightening (*68*–*70*), noting that robust global climate action is the only solution to limit the frequency and severity of exposure to heat stress.

## MATERIALS AND METHODS

### Dynamical downscaling using the near-global 10-km OFAM3 model

This work was delivered by the Commonwealth Scientific and Industrial Research Organization (CSIRO) Ocean Downscaling Strategic Project, which uses a three-dimensional ocean general circulation model, the OFAM3 (*48*), to downscale the future climate (*49*). The OFAM3 model has been extensively validated (*48*) and is used by the Australian Bureau of Meteorology to produce Australia’s operational ocean forecasts (*71*).

OFAM3 is a configuration of the Geophysical Fluid Dynamics Laboratory Modular Ocean Model version 4.1 (*72*), with 0.1° horizontal resolution (~10 km) for all longitudes and between 75°S and 75°N (*48*). OFAM3 has a fine vertical resolution in the top 200 m, with 5-m vertical resolution down to 20 m in depth and 10-m vertical resolution to 200 m in depth. There are 9 layers between 200 and 500 m in depth, 6 layers between 500 and 1000 m, and 12 layers below 1000 m.

OFAM3 was spun up by using repeat-year 1979 forcing from the Japanese 55-year Reanalysis (JRA-55) (*73*). Surface forcing was calculated with bulk formula. The atmospheric conditions include air temperature at 2 m in height, specific humidity at 2 m in height, eastward and northward wind speed at 10 m, shortwave downwelling radiation, longwave downwelling radiation, and precipitation. The historical downscaling experiment was forced with JRA-55 from 1979 to 2014. The SSS was weakly restored with a timescale of 180 days (*49*) to observed monthly climatology, the CSIRO Atlas for Regional Seas (*74*). The extensive validation details of the historical experiment are provided in (*49*).

The 21st century projections (2006 to 2101) were initialized from the end of 2005 of the historical experiment and driven by merged atmospheric conditions simulated by climate models and JRA-55. We briefly summarize the methodology here; the details were presented in the Supporting Information in (*50*). High-pass filtered JRA-55 (cut off at 7 years) is superimposed with climate change signals from 17 climate models (available at the time of experiments), following the approach in (*24*). The climate models are from the Climate Model Intercomparison Project Phase 5 (CMIP5) under the high-emission scenario RCP8.5 scenario, which represents the situation where emissions continue to grow and little action is taken to reduce emissions in the near future. The high-frequency variability from JRA-55 [frequency higher than 7 years to capture variability in the present-day climate such as the El Niño–Southern Oscillation (ENSO)] was combined with smoothed climate change signals (20-year running mean) to provide the forcing for the future simulation. The high-passed forcing over a 32-year period from 1981 to 2012 is repeated three times over 2006 to 2037, 2038 to 2069, and 2070 to 2101. The SSS was restored to observation-plus-climate-anomaly for future projections from 2006 to 2100 similar to the historical experiment.

Our approach assumed that seasonality in the future would be the same as in the present-day climate; hence, we are not imposing decadal variability to the forcing, and any projected changes in interannual variability are excluded. This is an ongoing research topic, given that there is limited agreement among climate models on even the most dominant interannual climate variability mode such as the ENSO, except some limited agreement on extremely strong ENSO events under future climate (*75*).

In this study, the ensemble mean of the 17 CMIP5 climate models is used to force the OFAM3 model to derive responses to climate change; therefore, changes associated with specific features are smoothed away. Previous studies have shown that the ensemble mean of climate models often produces the most robust signals given the limitations of individual climate models [e.g., (*47*, *76*)]. This approach is also a practical solution for global downscaling with limited computational resources, e.g., (*77*, *78*). A past study show that downscaled results driven by the ensemble mean fields are almost identical with the ensemble average results from individually downscaled cases, e.g., (*78*). Therefore, if only ensemble mean downscaled projections are desired, then an efficient alternative approach is to average the forcing changes first and then use the ensemble mean forcing for downscaling.

### Dynamical downscaling using the 4-km eReefs model

The eReefs model GBR4 is a three-dimensional ocean circulation model, which resolves key local processes such as tides (*79*). The GBR4 model is based on the Sparse Hydrodynamic Ocean Code, which has been extensively validated and used operationally by the eReefs project since September 2010 (*79*–*81*). The GBR4 model has an average horizontal resolution of ~4 km (180 × 600 cells) and a time step of 90 s (see Fig. 1). The high-resolution present-day climate simulation is carried out by the eReefs modeling team using the GBR4 model version 2.0 over 2010 to 2020 (*79*). GBR4 is nested within a global model of 10-km resolution, the Ocean Model, Analysis and Prediction System (http://wp.csiro.au/bluelink/global/oceanmaps/). GBR4 acts as a “bridging model” to a 1-km model, GBR1 (2839 × 510 cells), and both models represent the GBR domain.

The most relevant skill assessment for this study is upwelling events at the shelf break. In comparison to two offshore moorings, Benthuysen *et al.* found 25 of 53 events that have a skill score greater than 0.6 for seabed temperature (*37*). From available salinity data during these intrusions, skills scores were 0.82 (0.80) for near-surface (near-bottom) salinity (*37*, *82*). Further inshore skill assessments are undertaken in (*82*).

The future climate downscaling is carried out using the GBR4 model version 3.0 for two time slices (2050s and 2080s). The downscaling simulations were highly time-consuming and computationally expensive, so only two select decades were simulated: one in midcentury (2050s) and one toward the end of the century (2080s). OFAM3 provides the boundary conditions for the GBR4 model to account for the impact of large-scale circulation changes on the GBR. The future downscaling projections were carried out under negligible freshwater inputs.

### The treatment of tide in the present and future climate

The tide in the eReefs model is introduced by forcing elevation with 22 constituents derived from the Center for Space Research tidal model (*83*) and validated against 20 tide gauges along the coast. At the time of development of the model, we had no access to any future tidal model perturbed by climate change; therefore, we used current tidal constituent amplitudes and phases in future scenarios.

Mawson *et al.* (*55*) investigated the influence of sea level rise on tidal dynamics in coral reef systems using an unstructured numerical model of the GBR. Their study focuses on the impact on major tidal constituents and tidal range. Under three sea level rise scenarios, they found that the M2 constituent, the principal lunar semidiurnal tide, generally decreases across gauge locations as sea level rises, while the S3 constituent, the principal solar semidiurnal tide, generally increases across gauge locations. The O1 constituent, the principal lunar diurnal tide, and K1 constituent, the principal solar diurnal tide, display small variations or no change. The tidal range experiences up to 10% variation dependent on gauge locations. Harker *et al.* (*56*) and Schindelegger *et al.* (*57*) also found a complex nonlinear response pattern of M2 to sea level rise for NW Australia similar to Mawson *et al.* (*55*).

There might be changes in resonance due to sea level rise, which has not been studied so far when we performed a literature review. If sea level rise changes the wavelength required for resonance, then coastal amplitudes might change substantially if a resonance happened to be excited. This topic, however, is beyond the scope of this study.

### The bifurcation latitude of the SEC

The SEC bifurcation latitude is defined as the latitude of meridional current averaged over 1° (100 km) from the shelf break (i.e., 200-m isobath) to the open ocean is zero. For the depth-averaged annual SEC bifurcation latitude (fig. S9), we used the vertical average over the top 50 m, while earlier studies using coarse-resolution model/data typically calculated the average bifurcation latitude averaged over the top 200 m and 2° from the shelf break to the open ocean (*54*, *84*).

### The 2-km SSTAARS

We use a fine spatial-scale (~2 km) SST climatology constructed for the Australasia region, the SSTAARS (*28*). The climatology was constructed using 25 years (1992 to 2016) of Advanced Very High Resolution Radiometer (AVHRR) data from the National Oceanic and Atmosheric Administration (NOAA) Polar Orbiting Environmental Satellites (NPOES). The data were acquired by six Australian and two Antarctica reception stations from the AVHRR. The data have been processed following international Group for High Resolution SST protocols to help reduce instrument bias using in situ data. To reduce diurnal bias and cloud contamination, only nighttime nearly cloud-free data were used. The fine-scale SSTAARS climatology and associated statistics provide the best available SST regional climatology around Australia.

The high spatial resolution of 2 km for the SSTAARS was achieved through the use of real-time High Resolution Picture Transmission download from all operational NPOES during a satellite overpass reception stations acquiring data at 1.1-km resolution at nadir. These data provide a unique dataset back to 21 March 1992 (*28*). The SST data were bias corrected using in situ data and composited over a 2-km by 2-km grid. In contrast, because of data transfer and on-satellite data storage limitations, only low spatial resolution (4.4 km at nadir) AVHRR data were collected globally by these NPOES missions, which resulted in global-scale SST products before 2002 being limited at best to 5-km resolution (*85*, *86*).

### The CoralTemp 5-km SST dataset

The NOAA CRW program’s CoralTemp SST dataset (version 3.1) is a combination of two SST reanalysis products (1985 to 2016) and near-real-time satellite SST from October 2016 to present at 5-km resolution [see (*29*) for details]. This daily SST product is used to derive the CRW Coral Bleaching Heat Stress product suite, such as the Coral Bleaching HotSpot (HS) product and the DHW product. The HS is the difference between the daily SST and the MMM at each grid point. The DHW is the daily summation, over a 12-week (84 days) running window, of HS values of 1 or more, expressed as degrees Celsius weeks (°C-weeks). The DHW is a measure of accumulated daily heat stress, which has been shown to correlate strongly with coral bleaching, e.g., (*2*, *87*).

## Supplementary Materials

### This PDF file includes:

Figs. S1 to S11 References
