# Supplementary material for: Climate refugia in the Great Barrier Reef may endure into the future
Source: Sci Adv. 2024 Nov 29;10(48):eado6884. doi: 10.1126/sciadv.ado6884 (PMC11606497; doi:10.1126/sciadv.ado6884)
Supplement: Supplementary file 1 — Figs. S1 to S11 References [file sciadv.ado6884_sm.pdf]

Science Advances
AAAS

Supplementary Materials for

Climate refugia in the Great Barrier Reef may endure into the future

Chaojiao Sun *et al.*

Corresponding author: Chaojiao Sun, Chaojiao.Sun@csiro.au

Sci. Adv. **10**, eado6884 (2024)
DOI: 10.1126/sciadv.ado6884

This PDF file includes:

Figs. S1 to S11
References

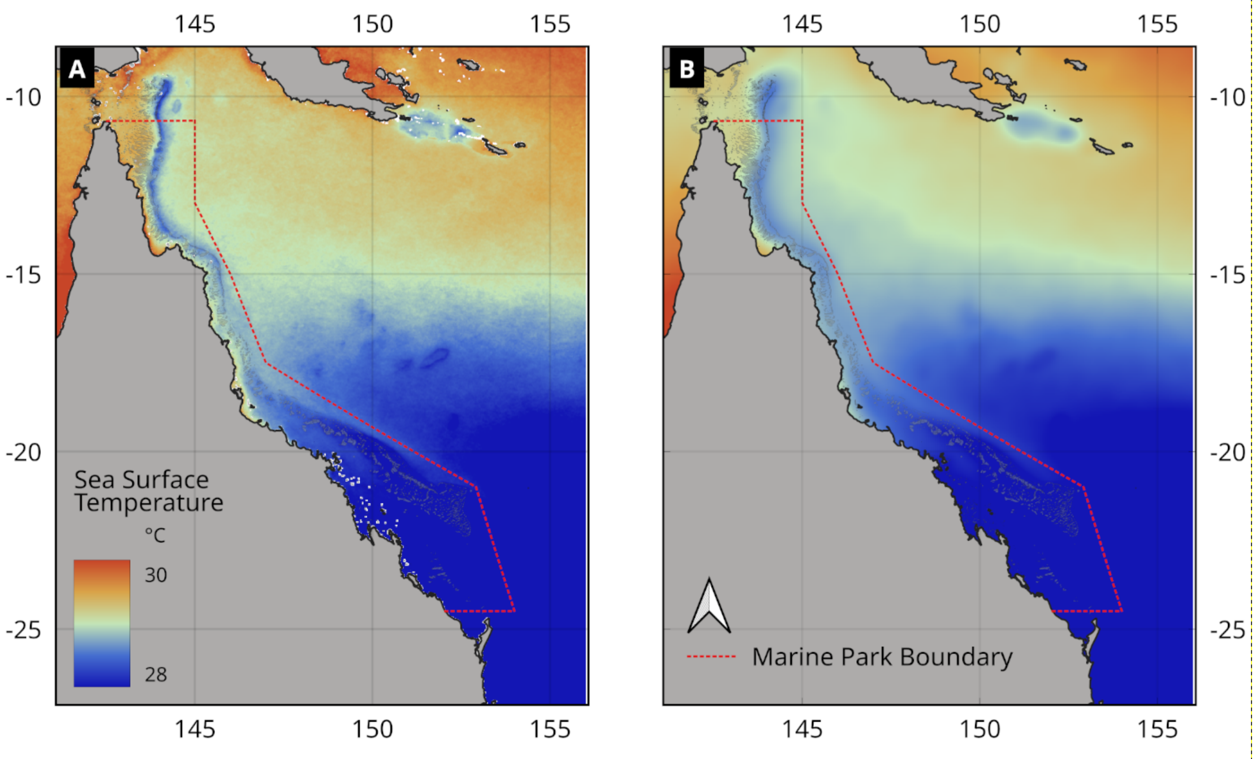

Fig. S1. Persistent upwelling in the austral summer season (January to March) demonstrated by two satellite SST datasets. (A) High-resolution (2 km) summer SST climatology over the summer from Sea Surface Temperature Atlas of the Australian Regional Seas (SSTAARS) (*28*). **(B)** NOAA 5 km resolution summer SST climatology from Coral Reef Watch (*29*). The dotted pink lines denote the GBR Marine Park boundary**.**

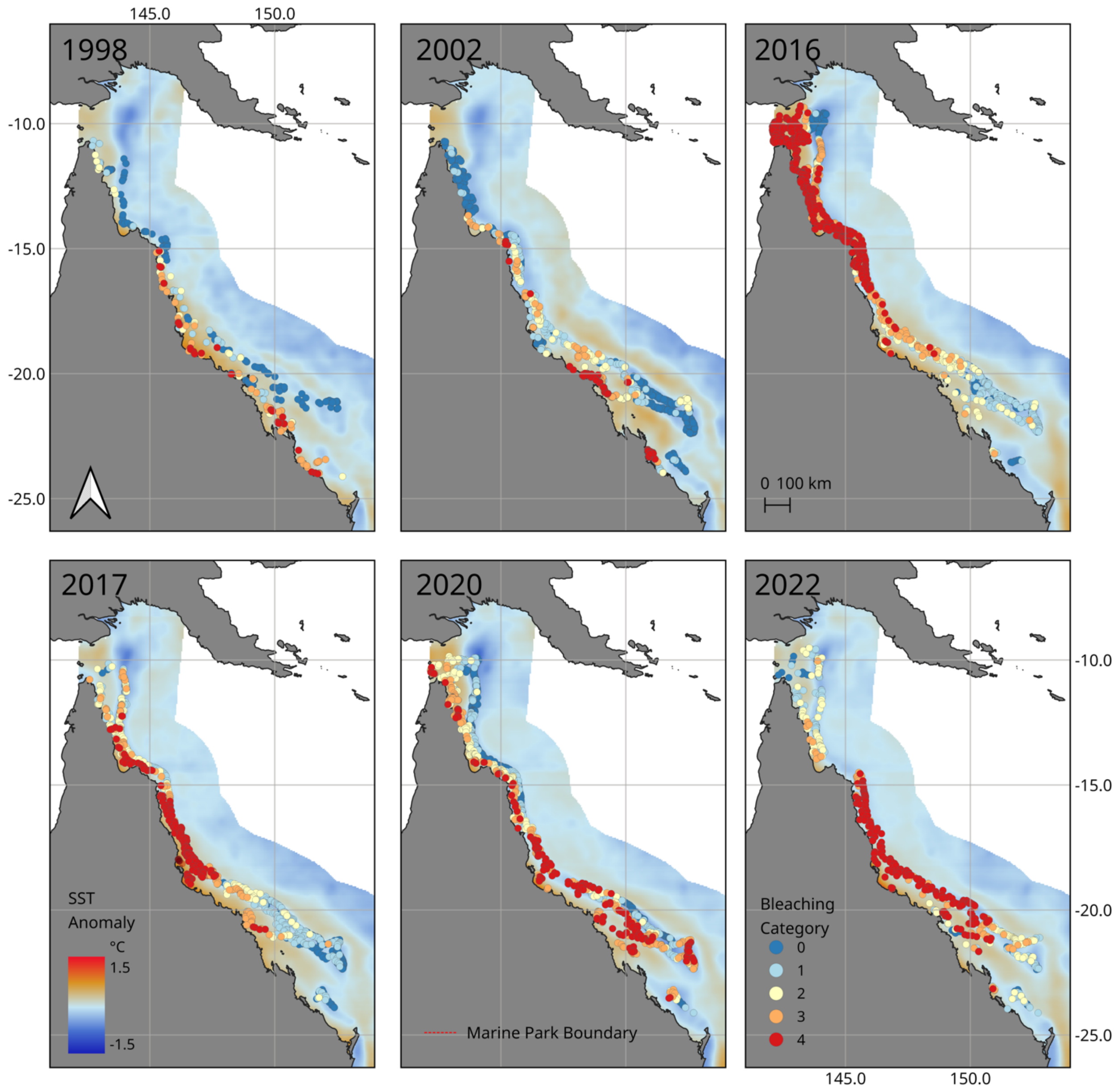

Fig. S2. The spatial extent and reef-level prevalence of shallow-water (< 6m) coral bleaching during six consecutive mass bleaching events on the Great Barrier Reef, with seasonal SST anomaly (January to March) from the NOAA Coral Reef Watch represented by color shadings. Aerial survey results are the same as in Fig. 1. The bleaching scores for the bleaching events in 1998, 2002, 2016, 2017, 2020, and 2022 are: blue (< 1% of shallow-water corals bleached), light blue (1–10%), yellow (10–30%), orange (30–60%), red (> 60%). The number of reefs surveyed each year was 587 in 1998, 630 in 2002, 1135 in 2016, 742 in 2017, 1036 in 2020, and 719 in 2022.

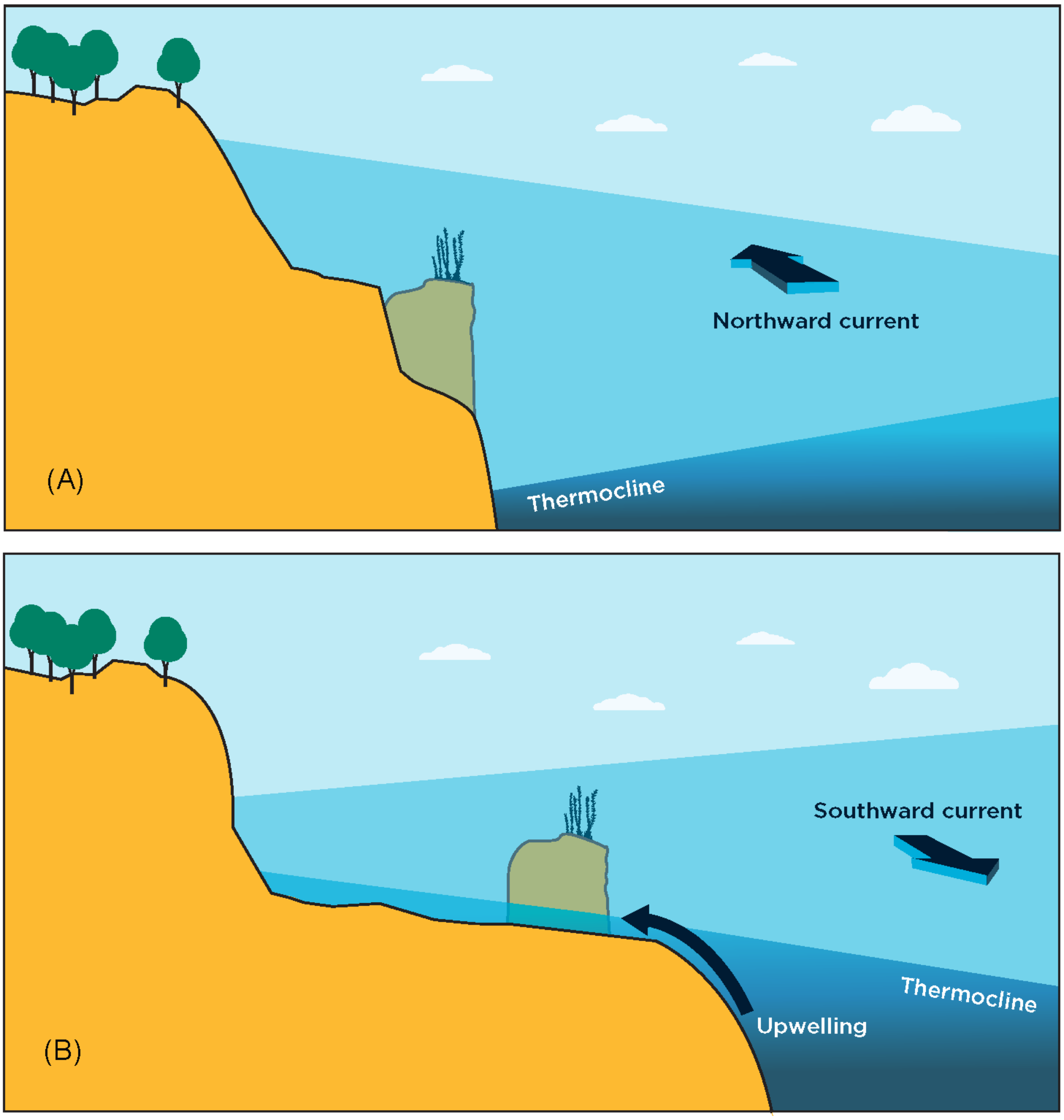

Fig. S3. Schematic showing cross sections of Gulf of Papua Current (GPC) and East Australian Current (EAC) with thermocline adjustment due to geostrophy (the balance of pressure gradient by the Coriolis force). (A) Along the GPC cross section, sea level rises and the thermocline deepens towards the coast. **(B)** Along the EAC cross section, sea level drops and the thermocline rises toward the coast, which facilitates upwelling of deeper water onto the continental shelf.

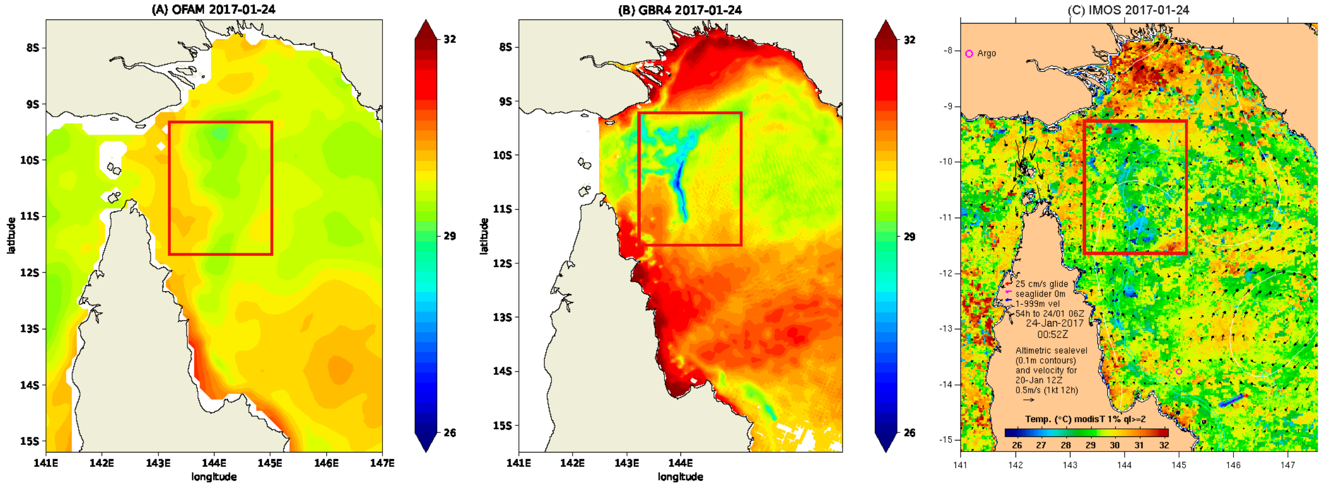

Fig. S4. An example of upwelling in the far northern GBR simulated by GBR4 and observed by satellites in comparison with OFAM3. (A) OFAM3 SST, **(B)** GBR4 SST, and **(C)** satellite SST provided by the Australian Integrated Marine Observing System (IMOS) OceanCurrent SST product, which was derived from multiple sensors from multiple satellites (*42*). All SSTs are shown for January 24, 2017. The IMOS OceanCurrent SST image is available at https://oceancurrent.aodn.org.au/sst.php?link=NGBR/2017/2017012400.html**.** The upwelling was well simulated by the GBR4 model (cool surface water in blue) but not the OFAM3 model. The GBR4 output (*79*) are available at https://thredds.nci.org.au/thredds/catalog/fx3/gbr4_v2/catalog.html, and OFAM3 output (*89*) are available at https://thredds.nci.org.au/thredds/catalog/gb6/BRAN/BRAN2020/catalog.html.

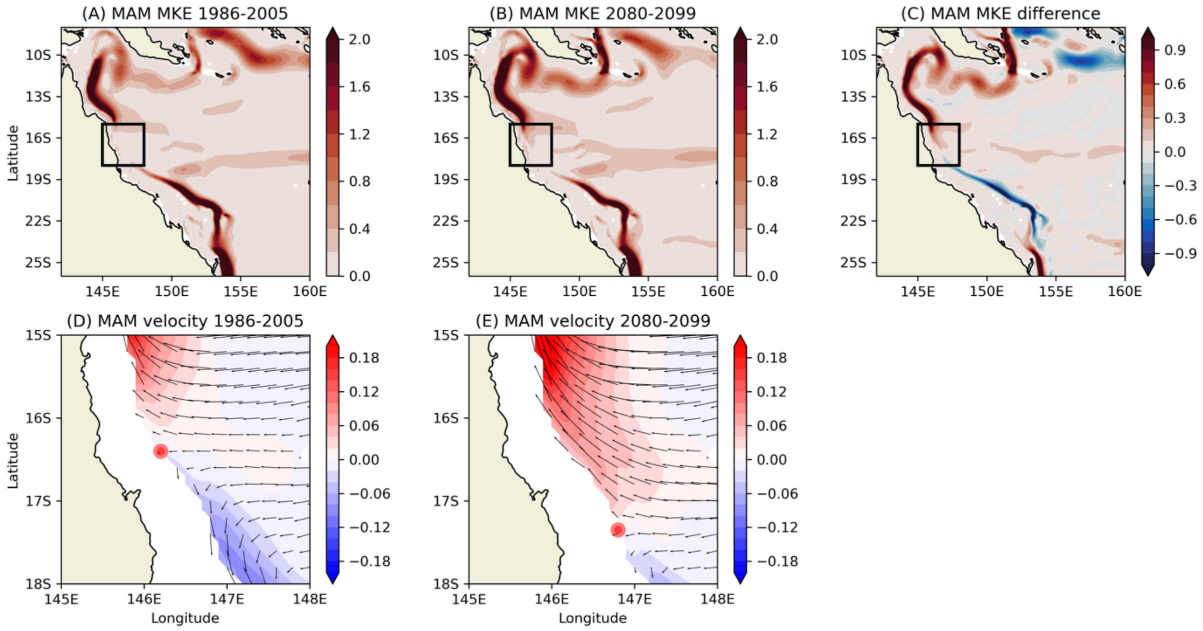

Fig. S5. Changes in strengths of ocean currents and SEC bifurcation location in the future for the austral autumn season March-April-May (MAM). Same as Fig. 5 except for MAM. Annual mean (over 20 years) of mean kinetic energy (MKE) (m^2/s^2) integrated over the top 50 m in **(A)** present climate (1986-2005) and **(B)** future climate (2080-2099). **(C)** difference in MKE between the future and present climate. **(D, E)** mean surface velocity (arrows) and meridional surface velocity (m/s) (colors). Note that the SEC bifurcation latitude in the present and future climate is marked by a red dot in (D) and (E). The extent of the plot in (D,E) is denoted by the rectangles in (A-C). Arrows in (D) and (E) denote the current direction and speed, while colors show meridional velocity with the transition from blue to red indicating the SEC bifurcation.

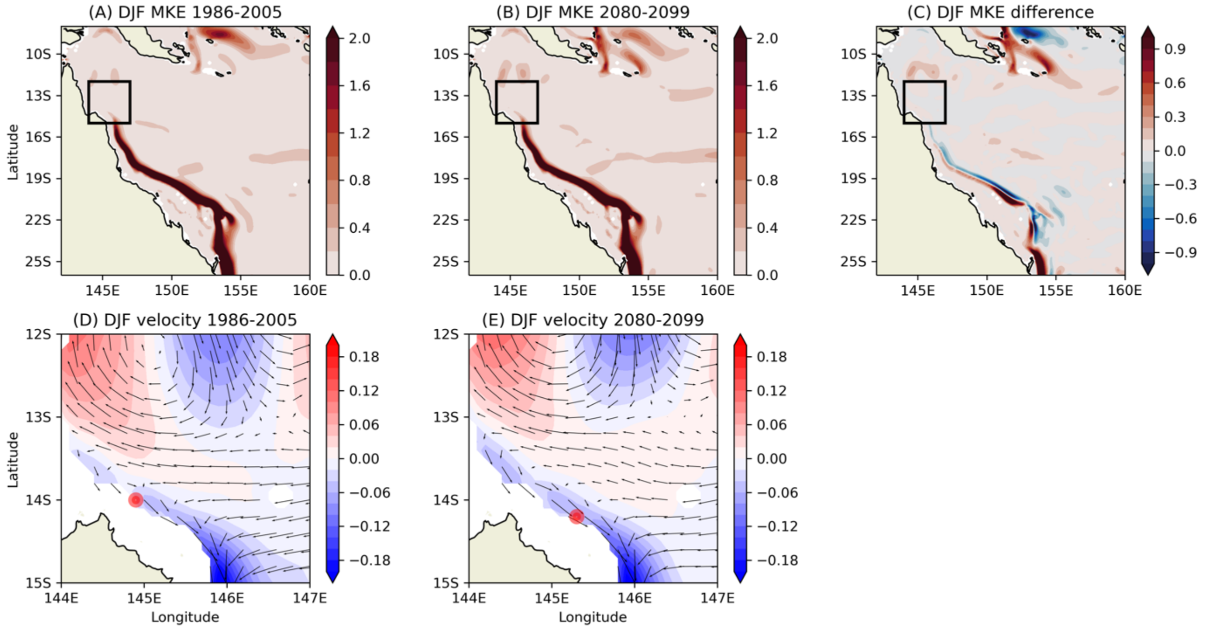

Fig. S6. Same as Fig. S5 but for the austral summer season December-January-February (DJF).

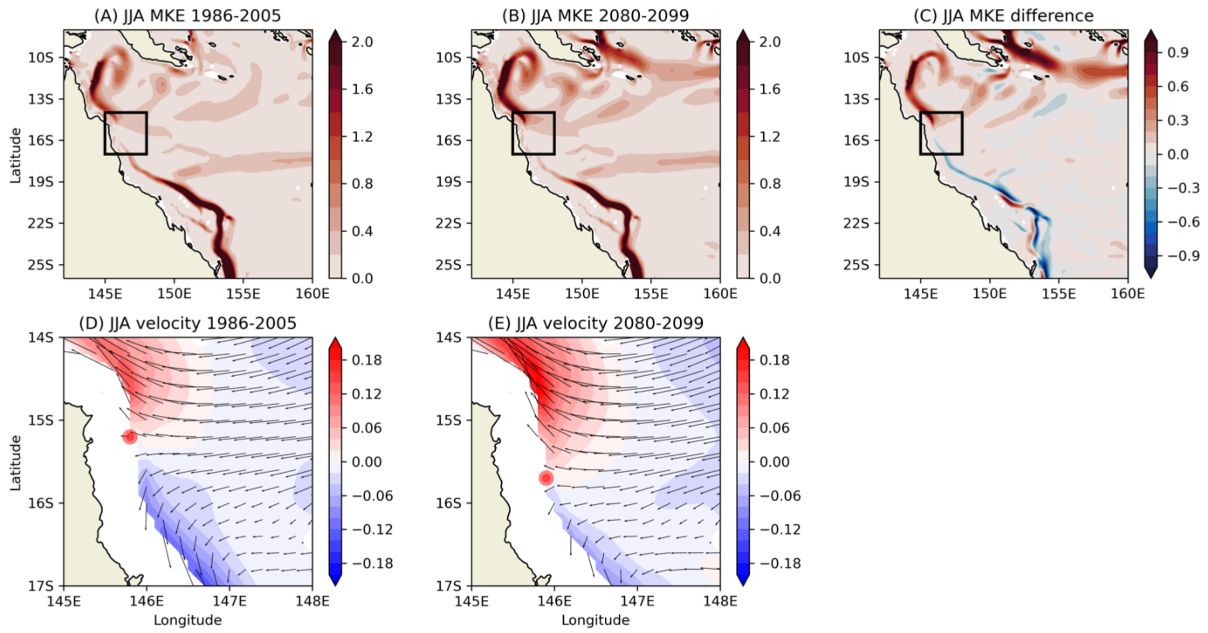

Fig. S7. Same as Fig. S5 but for the austral winter season June-July-August (JJA).

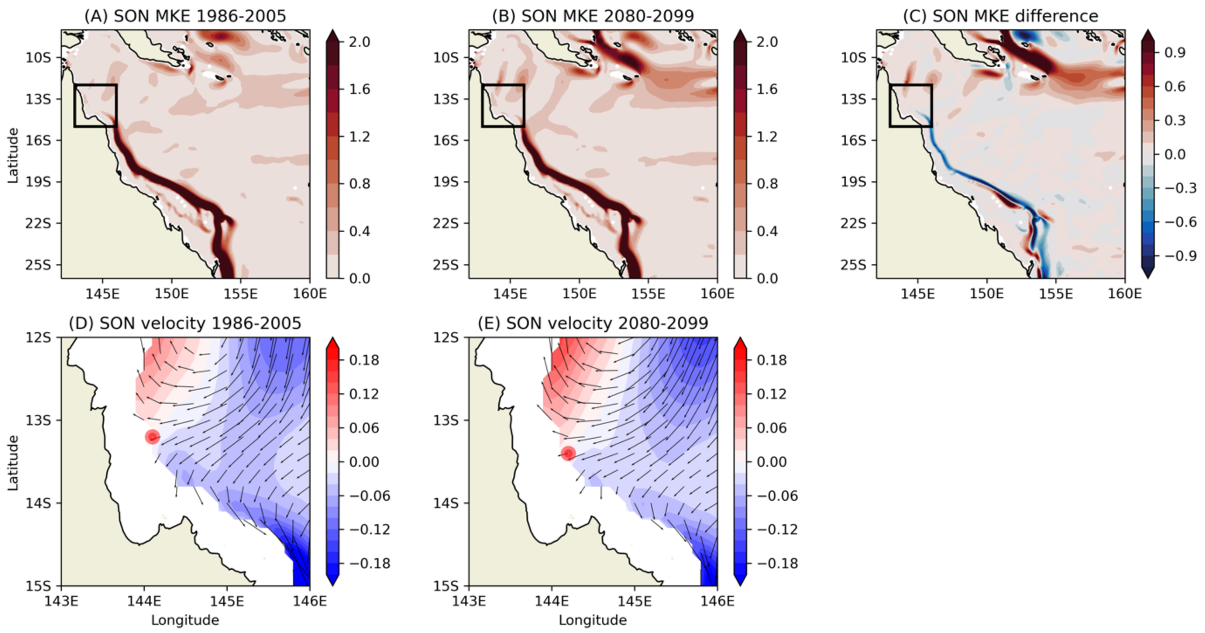

Fig. S8. Same as Fig. S5 but for the austral spring season September-October-November (SON).

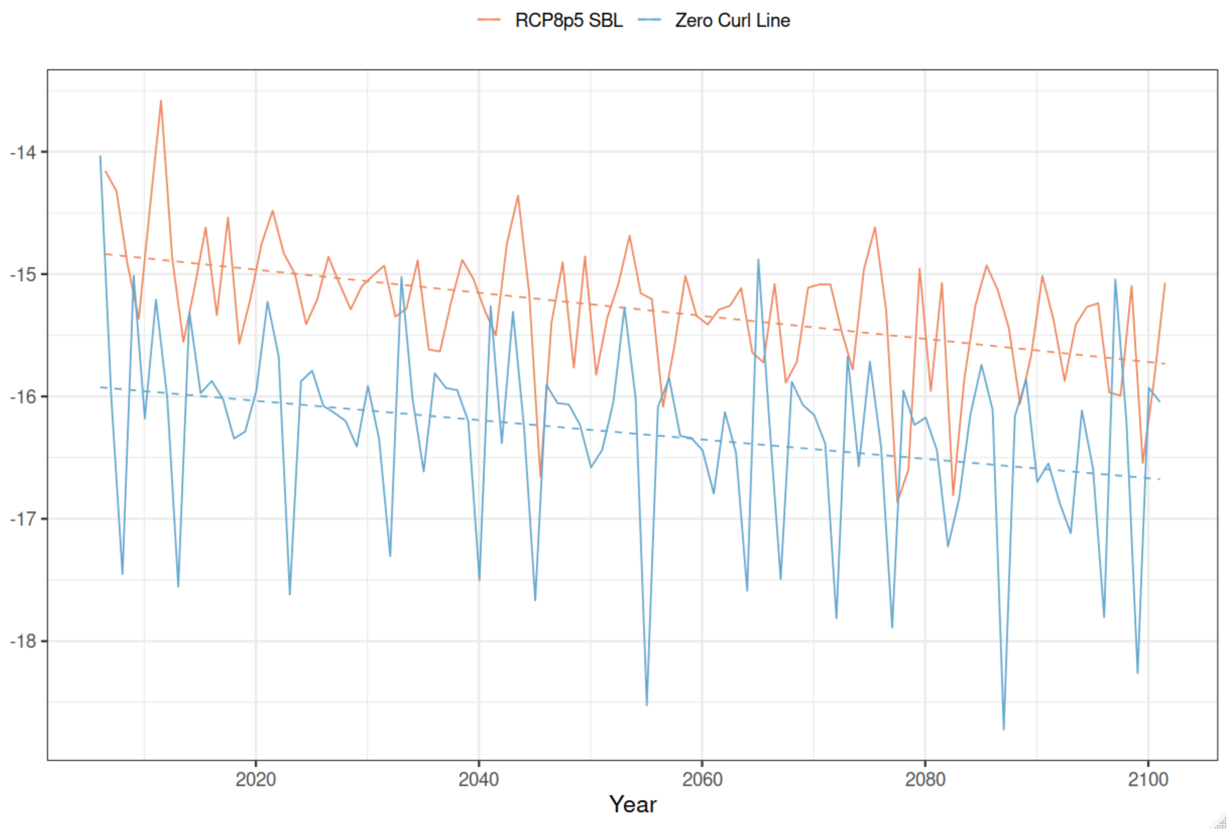

Fig. S9. The correspondence between SEC bifurcation latitude (annual averages) (in orange) and the latitude of zero wind stress curl integrated over the Pacific Ocean basin (in blue) over 2006-2101. The dashed lines are the trends over the century, showing a clear signal of southward shifts in both variables.

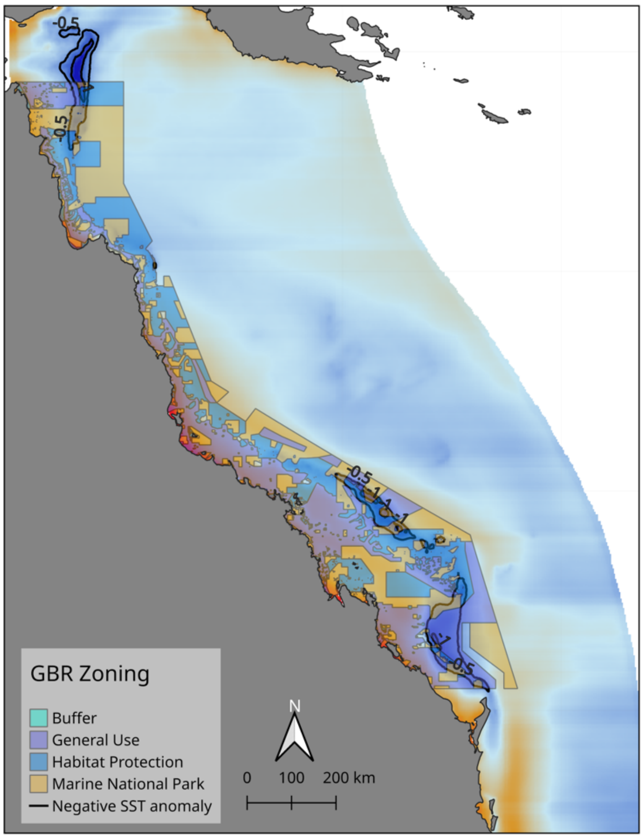

Fig. S10. Climate refugia in the present climate and its relationship with the GBR Marine Park Authority's designated zoning. The SST anomaly contour intervals are 0.5°C.

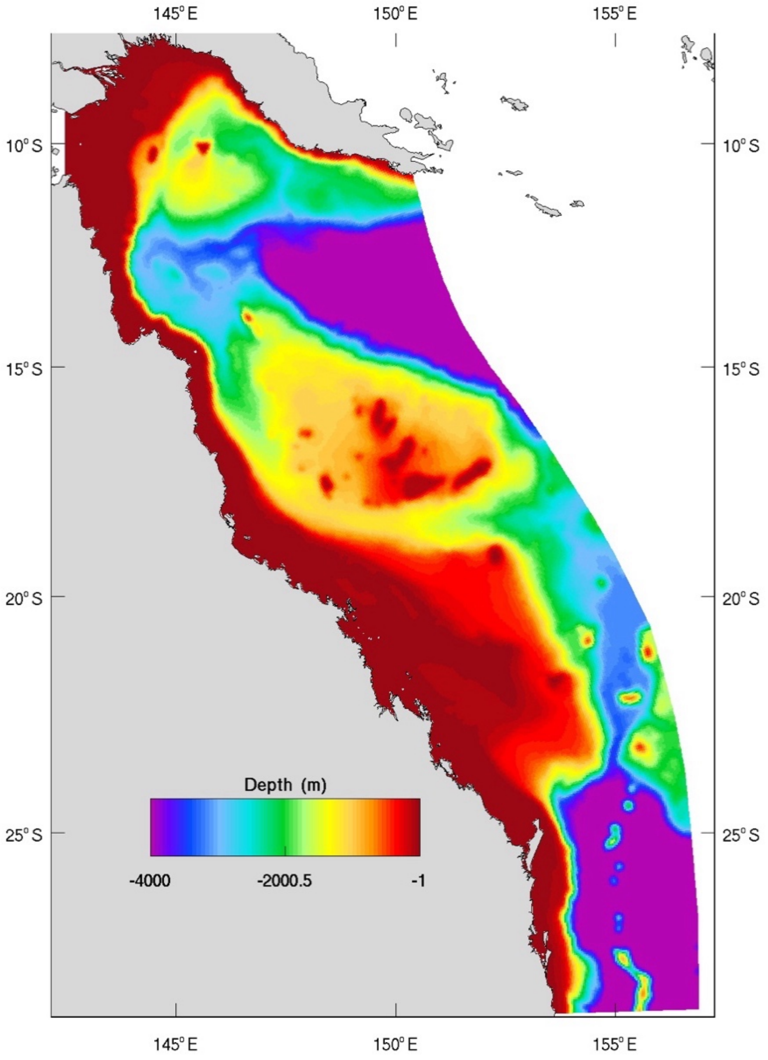

Fig. S11. Domain size and bathymetry of the GBR4 model.

REFERENCES AND NOTES

1. O. Hoegh-Guldberg, Climate change, coral bleaching and the future of the world's coral reefs. *Mar. Freshw. Res.* **50**, 839–866 (1999).

2. T. P. Hughes, J. T. Kerry, M. Álvarez-Noriega, J. G. Álvarez-Romero, K. D. Anderson, A. H. Baird, R. C. Babcock, M. Beger, D. R. Bellwood, R. Berkelmans, T. C. Bridge, I. R. Butler, M. Byrne, N. E. Cantin, S. Comeau, S. R. Connolly, G. S. Cumming, S. J. Dalton, G. Diaz-Pulido, C. M. Eakin, W. F. Figueira, J. P. Gilmour, H. B. Harrison, S. F. Heron, A. S. Hoey, J.-P. A. Hobbs, M. O. Hoogenboom, E. V. Kennedy, C.-y. Kuo, J. M. Lough, R. J. Lowe, G. Liu, M. T. McCulloch, H. A. Malcolm, M. J. McWilliam, J. M. Pandolfi, R. J. Pears, M. S. Pratchett, V. Schoepf, T. Simpson, W. J. Skirving, B. Sommer, G. Torda, D. R. Wachenfeld, B. L. Willis, S. K. Wilson, Global warming and recurrent mass bleaching of corals. *Nature* **543**, 373–377 (2017).

3. G. De'ath, K. E. Fabricius, H. Sweatman, M. Puotinen, The 27-year decline of coral cover on the Great Barrier Reef and its causes. *Proc. Natl. Acad. Sci. U.S.A.* **109**, 17995–17999 (2012).

4. S. F. Heron, J. A. Maynard, R. van Hooidonk, C. M. Eakin, Warming trends and bleaching stress of the world's coral reefs 1985–2012. *Sci. Rep.* **6**, 38402 (2016).

5. C. E. Langlais, A. Lenton, S. F. Heron, C. Evenhuis, A. Sen Gupta, J. N. Brown, M. Kuchinke, Coral bleaching pathways under the control of regional temperature variability. *Nat. Clim. Change* **7**, 839–844 (2017).

6. R. van Hooidonk, J. Maynard, J. Tamelander, J. Gove, G. Ahmadia, L. Raymundo, G. Williams, S. F. Heron, S. Planes, Local-scale projections of coral reef futures and implications of the Paris Agreement. *Sci. Rep.* **6**, 39666 (2016).

7. J. N. Smith, M. Mongin, A. Thompson, M. J. Jonker, G. De'ath, K. E. Fabricius, Shifts in coralline algae, macroalgae, and coral juveniles in the Great Barrier Reef associated with present-day ocean acidification. *Glob. Change Biol.* **26**, 2149–2160 (2020).

8. E. P. Lawrey, M. Stewart, "Mapping the Torres Strait Reef and Island Features: Extending the GBRFeatures (GBRMPA) dataset", Report to the National Environmental Science Programme (Reef and Rainforest Research Centre Limited, Cairns, 2016), p. 103.

9. R. M. Venegas, J. Acevedo, E. A. Treml, Three decades of ocean warming impacts on marine ecosystems: A review and perspective. *Deep Sea Res. 2 Top. Stud. Oceanogr.* **212**, 105318 (2023).

10. L. Cheng, J. Abraham, Z. Hausfather, K. E. Trenberth, How fast are the oceans warming? *Science* **363**, 128–129 (2019).

11. L. Cheng, J. Abraham, K. E. Trenberth, J. Fasullo, T. Boyer, R. Locarnini, B. Zhang, F. Yu, L. Wan, X. Chen, X. Song, Y. Liu, M. E. Mann, F. Reseghetti, S. Simoncelli, V. Gouretski, G. Chen, A. Mishonov, J. Reagan, J. Zhu, Upper ocean temperatures hit record high in 2020. *Adv. Atmos. Sci.* **38**, 523–530 (2021).

12. IPCC, Summary for Policymakers, in *IPCC Special Report on the Ocean and Cryosphere in a Changing Climate*, H.-O. Pörtner, D. C. Roberts, V. Masson-Delmotte, P. Zhai, M. Tignor, E. Poloczanska, K. Mintenbeck, A. Alegría, M. Nicolai, A. Okem, J. Petzold, B. Rama, N. M. Weyer, Eds. (Cambridge Univ. Press, 2019).

13. A. H. Baird, J. S. Madin, M. Álvarez-Noriega, L. Fontoura, J. T. Kerry, C. Y. Kuo, K. Precoda, D. Torres-Pulliza, R. M. Woods, K. J. A. Zawada, T. P. Hughes, A decline in bleaching suggests that depth can provide a refuge from global warming in most coral taxa. *Mar. Ecol. Prog. Ser.* **603**, 257–264 (2018).

14. C. M. Eakin, H. P. A. Sweatman, R. E. Brainard, The 2014–2017 global-scale coral bleaching event: Insights and impacts. *Coral Reefs* **38**, 539–545 (2019).

15. K. B. Karnauskas, A. L. Cohen, Equatorial refuge amid tropical warming. *Nat. Clim. Change* **2**, 530–534 (2012).

16. J. Kavousi, G. Keppel, Clarifying the concept of climate change refugia for coral reefs. *ICES J. Mar. Sci.* **75**, 43–49 (2018).

17. R. O. R. Y. Thompson, T. J. Golding, Tidally induced ‘upwelling’ by the Great Barrier Reef. *J. Geophys. Res. Oceans* **86**, 6517–6521 (1981).

18. M. D. Fox, R. Guillaume-Castel, C. B. Edwards, J. Glanz, J. M. Gove, J. A. M. Green, E. Juhlin, J. E. Smith, G. J. Williams, Ocean currents magnify upwelling and deliver nutritional subsidies to reef-building corals during El Niño heatwaves. *Sci. Adv.* **9**, eaad5032 (2023).

19. P. Izquierdo, F. G. Taboada, R. González-Gil, J. Arrontes, J. M. Rico, Alongshore upwelling modulates the intensity of marine heatwaves in a temperate coastal sea. *Sci. Total Environ.* **835**, 155478 (2022).

20. J. Figueiredo, C. J. Thomas, E. Deleersnijder, J. Lambrechts, A. H. Baird, S. R. Connolly, E. Hanert, Global warming decreases connectivity among coral populations. *Nat. Clim. Change* **12**, 83–87 (2022).

21. A. M. Dixon, P. M. Forster, S. F. Heron, A. M. K. Stoner, M. Beger, Future loss of local-scale thermal refugia in coral reef ecosystems. *PLOS Clim.* **1**, e0000004 (2022).

22. J. K. McWhorter, P. R. Halloran, G. Roff, W. J. Skirving, P. J. Mumby, Climate refugia on the Great Barrier Reef fail when global warming exceeds 3°C. *Glob. Change Biol.* **28**, 5768–5780 (2022).

23. R. van Hooidonk, J. A. Maynard, Y. Liu, S.-K. Lee, Downscaled projections of Caribbean coral bleaching that can inform conservation planning. *Glob. Change Biol.* **21**, 3389–3401 (2015).

24. C. Sun, M. Feng, R. J. Matear, M. A. Chamberlain, P. Craig, K. R. Ridgway, A. Schiller, Marine downscaling of a future climate scenario for australian boundary currents. *J. Clim.* **25**, 2947–2962 (2012).

25. C. R. Schwalm, S. Glendon, P. B. Duffy, RCP8.5 tracks cumulative CO_2 emissions. *Proc. Natl. Acad. Sci. U.S.A.* **117**, 19656–19657 (2020).

26. V. Oerder, J. Bento, C. Morales, S. Hormazabal, O. Pizarro, Coastal upwelling front detection off central Chile (36.5–37°S) and spatio-temporal variability of frontal characteristics. *Remote Sens.* **10**, 690 (2018).

27. G. Liu, C. M. Eakin, M. Chen, A. Kumar, J. L. De La Cour, S. F. Heron, E. F. Geiger, W. J. Skirving, K. V. Tirak, A. E. Strong, Predicting heat stress to inform reef management: NOAA Coral Reef Watch's 4-month coral bleaching outlook. *Front. Mar. Sci.* **5**, 57 (2018).

28. S. E. Wijffels, H. Beggs, C. Griffin, J. F. Middleton, M. Cahill, E. King, E. Jones, M. Feng, J. A. Benthuysen, C. R. Steinberg, P. Sutton, A fine spatial-scale sea surface temperature atlas of the Australian regional seas (SSTAARS): Seasonal variability and trends around Australasia and New Zealand revisited. *J. Mar. Syst.* **187**, 156–196 (2018).

29. W. Skirving, B. Marsh, J. De La Cour, G. Liu, A. Harris, E. Maturi, E. Geiger, C. M. Eakin, CoralTemp and the coral reef watch coral bleaching heat stress product suite version 3.1. *Remote Sens.* **12**, 3856 (2020).

30. T. P. Hughes, J. T. Kerry, S. R. Connolly, J. G. Álvarez-Romero, C. M. Eakin, S. F. Heron, M. A. Gonzalez, J. Moneghetti, Emergent properties in the responses of tropical corals to recurrent climate extremes. *Curr. Biol.* **31**, 5393–5399.e3 (2021).

31. R. Berkelmans, G. Death, S. Kininmonth, W. J. Skirving, A comparison of the 1998 and 2002 coral bleaching events on the Great Barrier Reef: Spatial correlation, patterns, and predictions. *Coral Reefs* **23**, 74–83 (2004).

32. T. P. Hughes, J. T. Kerry, A. H. Baird, S. R. Connolly, A. Dietzel, C. M. Eakin, S. F. Heron, A. S. Hoey, M. O. Hoogenboom, G. Liu, M. J. McWilliam, R. J. Pears, M. S. Pratchett, W. J. Skirving, J. S. Stella, G. Torda, Global warming transforms coral reef assemblages. *Nature* **556**, 492–496 (2018).

33. N. E. Cantin, E. Klein-Salas, P. Frade, "Spatial variability in coral bleaching severity and mortality during the 2016 and 2017 Great Barrier Reef coral bleaching events", Report to the National Environmental Science Program (Reef and Rainforest Research Centre Limited, Cairns 2021), p. 64.

34. E. Wolanski, *Physical Oceanographic Processes of the Great Barrier Reef* (CRC Press, ed. 1, 1994).

35. P. J. Durack, S. E. Wijffels, R. J. Matear, Ocean salinities reveal strong global water cycle intensification during 1950 to 2000. *Science* **336**, 455–458 (2012).

36. J. C. Andrews, M. J. Furnas, Subsurface intrusions of Coral Sea water into the central Great Barrier Reef—I. Structures and shelf-scale dynamics. *Cont. Shelf Res.* **6**, 491–514 (1986).

37. J. A. Benthuysen, H. Tonin, R. Brinkman, M. Herzfeld, C. Steinberg, Intrusive upwelling in the Central Great Barrier Reef. *J. Geophys. Res. Oceans* **121**, 8395–8416 (2016).

38. S. J. Weeks, A. Bakun, C. R. Steinberg, R. Brinkman, O. Hoegh-Guldberg, The Capricorn Eddy: A prominent driver of the ecology and future of the southern Great Barrier Reef. *Coral Reefs* **29**, 975–985 (2010).

39. S. Condie, R. Condie, Retention of plankton within ocean eddies. *Glob. Ecol. Biogeogr.* **25**, 1264–1277 (2016).

40. A. S. J. Wyatt, J. J. Leichter, L. T. Toth, T. Miyajima, R. B. Aronson, T. Nagata, Heat accumulation on coral reefs mitigated by internal waves. *Nat. Geosci.* **13**, 28–34 (2020).

41. C.R. Steinberg, J. Benthuysen, E. Klein-Salas, N.E. Cantin, H. Tonin, S. Spagnol, C.M. Spillman, "Oceanographic drivers of bleaching in the GBR: from observations to prediction, Volume 1: Summary ofoceanographic conditions during the 2015-17 bleaching years", Report to the National Environmental Science Program (Reef and Rainforest Research Centre Limited, Cairns 2021), p. 52.

42. P. D. Govekar, C. Griffin, H. Beggs, Multi-sensor sea surface temperature products from the Australian Bureau of Meteorology. *Remote Sens.* **14**, 3785 (2022).

43. S. A. Condie, Interactions between western boundary currents and shelf waters: A mechanism for coastal upwelling. *J. Geophys. Res. Oceans* **100**, 24811–24818 (1995).

44. W. S. Kessler, S. Cravatte, Mean circulation of the Coral Sea. *J. Geophys. Res. Oceans* **118**, 6385–6410 (2013).

45. A. Ganachaud, S. Cravatte, A. Melet, A. Schiller, N. J. Holbrook, B. M. Sloyan, M. J. Widlansky, M. Bowen, J. Verron, P. Wiles, K. Ridgway, P. Sutton, J. Sprintall, C. Steinberg, G. Brassington, W. Cai, R. Davis, F. Gasparin, L. Gourdeau, T. Hasegawa, W. Kessler, C. Maes, K. Takahashi, K. J. Richards, U. Send, The Southwest Pacific Ocean circulation and climate experiment (SPICE). *J. Geophys. Res. Oceans* **119**, 7660–7686 (2014).

46. K. Hock, N. H. Wolff, J. C. Ortiz, S. A. Condie, K. R. N. Anthony, P. G. Blackwell, P. J. Mumby, Connectivity and systemic resilience of the Great Barrier Reef. *PLOS Biol.* **15**, e2003355 (2017).

47. A. Sen Gupta, A. Stellema, G. M. Pontes, A. S. Taschetto, A. Vergés, V. Rossi, Future changes to the upper ocean Western Boundary Currents across two generations of climate models. *Sci. Rep.* **11**, 9538 (2021).

48. P. R. Oke, D. A. Griffin, A. Schiller, R. J. Matear, R. Fiedler, J. Mansbridge, A. Lenton, M. Cahill, M. A. Chamberlain, K. Ridgway, Evaluation of a near-global eddy-resolving ocean model. *Geosci. Model Dev.* **6**, 591–615 (2013).

49. X. Zhang, P. R. Oke, M. Feng, M. A. Chamberlain, J. A. Church, D. Monselesan, C. Sun, R. J. Matear, A. Schiller, R. Fiedler, A near-global eddy-resolving OGCM for climate studies. *Geosci. Model Dev. Discuss.* **2016**, 1–52 (2016).

50. X. Zhang, J. A. Church, D. Monselesan, K. L. McInnes, Sea level projections for the Australian region in the 21st century. *Geophys. Res. Lett.* **44**, 8481–8491 (2017).

51. D. Hu, L. Wu, W. Cai, A. S. Gupta, A. Ganachaud, B. Qiu, A. L. Gordon, X. Lin, Z. Chen, S. Hu, G. Wang, Q. Wang, J. Sprintall, T. Qu, Y. Kashino, F. Wang, W. S. Kessler, Pacific western boundary currents and their roles in climate. *Nature* **522**, 299–308 (2015).

52. K. R. Ridgway, J. A. Benthuysen, C. Steinberg, Closing the gap between the Coral Sea and the equator: Direct observations of the north Australian western boundary currents. *J. Geophys. Res. Oceans* **123**, 9212–9231 (2018).

53. W. S. Kessler, S. Cravatte, ENSO and short-term variability of the South Equatorial Current entering the Coral Sea. *J. Phys. Oceanogr.* **43**, 956–969 (2013).

54. F. Zhai, D. Hu, Q. Wang, F. Wang, Long-term trend of Pacific South Equatorial Current bifurcation over 1950–2010. *Geophys. Res. Lett.* **41**, 3172–3180 (2014).

55. E. E. Mawson, K. C. Lee, J. Hill, Sea level rise and the Great Barrier Reef: The future implications on reef tidal dynamics. *J. Geophys. Res. Oceans* **127**, e2021JC017823 (2022).

56. A. Harker, J. A. M. Green, M. Schindelegger, S. B. Wilmes, The impact of sea-level rise on tidal characteristics around Australia. *Ocean Sci.* **15**, 147–159 (2019).

57. M. Schindelegger, J. A. M. Green, S.-B. Wilmes, I. D. Haigh, Can we model the effect of observed sea level rise on tides? *J. Geophys. Res. Oceans* **123**, 4593–4609 (2018).

58. C. Frazão Santos, T. Agardy, F. Andrade, H. Calado, L. B. Crowder, C. N. Ehler, S. García-Morales, E. Gissi, B. S. Halpern, M. K. Orbach, H.-O. Pörtner, R. Rosa, Integrating climate change in ocean planning. *Nat. Sustain.* **3**, 505–516 (2020).

59. R. van Hooidonk, J. A. Maynard, S. Planes, Temporary refugia for coral reefs in a warming world. *Nat. Clim Change* **3**, 508–511 (2013).

60. I. Chollett, P. J. Mumby, J. Cortés, Upwelling areas do not guarantee refuge for coral reefs in a warming ocean. *Mar. Ecol. Prog. Ser.* **416**, 47–56 (2010).

61. Z. Huang, M. Feng, S. J. Dalton, A. G. Carroll, Marine heatwaves in the Great Barrier Reef and Coral Sea: Their mechanisms and impacts on shallow and mesophotic coral ecosystems. *Sci. Total Environ.* **908**, 168063 (2024).

62. D. J. Amaya, M. G. Jacox, M. R. Fewings, V. S. Saba, M. F. Stuecker, R. R. Rykaczewski, A. C. Ross, C. A. Stock, A. Capotondi, C. M. Petrik, S. J. Bograd, M. A. Alexander, W. Cheng,

A. J. Hermann, K. A. Kearney, B. S. Powell, Marine heatwaves need clear definitions so coastal communities can adapt. *Nature* **616**, 29–32 (2023).

63. A. J. Hobday, L. V. Alexander, S. E. Perkins, D. A. Smale, S. C. Straub, E. C. J. Oliver, J. A. Benthuysen, M. T. Burrows, M. G. Donat, M. Feng, N. J. Holbrook, P. J. Moore, H. A. Scannell, A. Sen Gupta, T. Wernberg, A hierarchical approach to defining marine heatwaves. *Prog. Oceanogr.* **141**, 227–238 (2016).

64. C. Mellin, S. Brown, N. Cantin, E. Klein-Salas, D. Mouillot, S. F. Heron, D. A. Fordham, Cumulative risk of future bleaching for the world's coral reefs. *Sci. Adv.* **10**, eadn9660 (2024).

65. R. C. Babcock, J. M. Dambacher, E. B. Morello, É. E. Plagányi, K. R. Hayes, H. P. A. Sweatman, M. S. Pratchett, Assessing different causes of crown-of-thorns starfish outbreaks and appropriate responses for management on the Great Barrier Reef. *PLOS ONE* **11**, e0169048 (2016).

66. S. A. Condie, É. E. Plagányi, E. B. Morello, K. Hock, R. Beeden, Great Barrier Reef recovery through multiple interventions. *Conserv. Biol.* **32**, 1356-1367 (2018).

67. S. A. Condie, K. R. N. Anthony, R. C. Babcock, M. E. Baird, R. Beeden, C. S. Fletcher, R. Gorton, D. Harrison, A. J. Hobday, É. E. Plagányi, D. A. Westcott, Large-scale interventions may delay decline of the Great Barrier Reef. *R Soc. Open. Sci.* **8**, 201296 (2021).

68. O. Hoegh-Guldberg, L. Hughes, S. McIntyre, D. B. Lindenmayer, C. Parmesan, H. P. Possingham, C. D. Thomas, Assisted Colonization and Rapid Climate Change. *Science* **321**, 345-346 (2008).

69. L. B. DeFilippo, L. C. McManus, D. E. Schindler, M. L. Pinsky, M. A. Colton, H. E. Fox, E. W. Tekwa, S. R. Palumbi, T. E. Essington, M. M. Webster, Assessing the potential for demographic restoration and assisted evolution to build climate resilience in coral reefs. *Ecol. Appl.* **32**, e2650, (2022).

70. J. Latham, K. Bower, T. Choularton, H. Coe, P. Connolly, G. Cooper, T. Craft, J. Foster, A. Gadian, L. Galbraith, H. Iacovides, D. Johnston, B. Launder, B. Leslie, J. Meyer, A. Neukermans, B. Ormond, B. Parkes, P. Rasch, J. Rush, S. Salter, T. Stevenson, H. Wang, Q. Wang, R. Wood, Marine cloud brightening. *Philos. Trans. A Math. Phys. Eng. Sci.* **370**, 4217-4262 (2012).

71. A. Schiller, G. B. Brassington, P. Oke, M. Cahill, P. Divakaran, M. Entel, J. Freeman, D. Griffin, M. Herzfeld, R. Hoeke, X. Huang, E. Jones, E. King, B. Parker, T. Pitman, U. Rosebrock, J. Sweeney, A. Taylor, M. Thatcher, R. Woodham, A. Zhong, Bluelink ocean forecasting Australia: 15 years of operational ocean service delivery with societal, economic and environmental benefits. *J. Oper. Oceanogr.* **13**, 1–18 (2020).

72. S. M. Griffies, "Elements of MOM4p1" (NOAA/Geophysical Fluid Dynamics Laboratory, 2009).

73. S. Kobayashi, Y. Ota, Y. Harada, A. Ebita, M. Moriya, H. Onoda, K. Onogi, H. Kamahori, C. Kobayashi, H. Endo, K. Miyaoka, K. Takahashi, The JRA-55 reanalysis: General specifications and basic characteristics. *J. Meteorol. Soc. Japan Ser. II* **93**, 5–48 (2015).

74. K. R. Ridgway, J. R. Dunn, Mesoscale structure of the mean East Australian Current System and its relationship with topography. *Prog. Oceanogr.* **56**, 189–222 (2003).

75. W. Cai, A. Santoso, M. Collins, B. Dewitte, C. Karamperidou, J.-S. Kug, M. Lengaigne, M. J. McPhaden, M. F. Stuecker, A. S. Taschetto, A. Timmermann, L. Wu, S.-W. Yeh, G. Wang, B. Ng, F. Jia, Y. Yang, J. Ying, X.-T. Zheng, T. Bayr, J. R. Brown, A. Capotondi, K. M. Cobb, B. Gan, T. Geng, Y.-G. Ham, F.-F. Jin, H.-S. Jo, X. Li, X. Lin, S. McGregor, J.-H. Park, K. Stein, K. Yang, L. Zhang, W. Zhong, Changing El Niño–Southern Oscillation in a warming climate. *Nat. Rev. Earth Environ.* **2**, 628–644 (2021).

76. A. Sen Gupta, A. Ganachaud, S. McGregor, J. N. Brown, L. Muir, Drivers of the projected changes to the Pacific Ocean equatorial circulation. *Geophys. Res. Lett.* **39**, L09605 (2012).

77. Y. Liu, S.-K. Lee, B. A. Muhling, J. T. Lamkin, D. B. Enfield, Significant reduction of the Loop Current in the 21st century and its impact on the Gulf of Mexico. *J. Geophys. Res. Oceans* **117**, C05039 (2012).

78. Y. Jin, X. Zhang, J. A. Church, X. Bao, Projected sea level changes in the marginal seas near China based on dynamical downscaling. *J. Clim.* **34**, 7037–7055 (2021).

79. A. D. L. Steven, M. E. Baird, R. Brinkman, N. J. Car, S. J. Cox, M. Herzfeld, J. Hodge, E. Jones, E. King, N. Margvelashvili, C. Robillot, B. Robson, T. Schroeder, J. Skerratt, S. Tickell, N. Tuteja, K. Wild-Allen, J. Yu, eReefs: An operational information system for managing the Great Barrier Reef. *J. Oper. Oceanogr.* **12**, S12–S28 (2019).

80. A. Schiller, M. Herzfeld, R. Brinkman, F. Rizwi, J. Andrewartha, Cross-shelf exchanges between the Coral Sea and the Great Barrier Reef lagoon determined from a regional-scale numerical model. *Cont. Shelf Res.* **109**, 150–163 (2015).

81. M. Herzfeld, J. Andrewartha, M. Baird, R. Brinkman, M. Furnas, P. Gillibrand, M. Hemer, K. Joehnk, E. Jones, D. McKinnon, N. Margvelashvili, M. Mongin, P. Oke, F. Rizwi, B. Robson, S. Seaton, J. Skerratt, H. Tonin, K. Wild-Allen, "eReefs Marine Modelling: Final Report" (CSIRO, 2016).

82. M. Herzfeld, P. A. Gillibrand, Active open boundary forcing using dual relaxation time-scales in downscaled ocean models. *Ocean Model.* **89**, 71–83 (2015).

83. D. E. Cartwright, R. D. Ray, Oceanic tides from Geosat altimetry. *J. Geophys. Res. Oceans* **95**, 3069–3090 (1990).

84. B. Qiu, S. Chen, Interannual-to-decadal variability in the bifurcation of the North Equatorial Current off the Philippines. *J. Phys. Oceanogr.* **40**, 2525–2538 (2010).

85. C. J. Merchant, O. Embury, J. Roberts-Jones, E. Fiedler, C. E. Bulgin, G. K. Corlett, S. Good, A. McLaren, N. Rayner, S. Morak-Bozzo, C. Donlon, Sea surface temperature datasets for climate applications from Phase 1 of the European Space Agency Climate Change Initiative (SST CCI). *Geosci. Data J.* **1**, 179–191 (2014).

86. J. Roberts-Jones, E. K. Fiedler, M. J. Martin, Daily, global, high-resolution SST and sea ice reanalysis for 1985–2007 using the OSTIA system. *J. Clim.* **25**, 6215–6232 (2012).

87. S. F. Heron, L. Johnston, G. Liu, E. F. Geiger, J. A. Maynard, J. L. De La Cour, S. Johnson, R. Okano, D. Benavente, T. F. R. Burgess, J. Iguel, D. I. Perez, W. J. Skirving, A. E. Strong, K. Tirak, C. M. Eakin, Validation of reef-scale thermal stress satellite products for coral bleaching monitoring. *Remote Sens.* **8**, 59 (2016).

88. T. P. Hughes, J. T. Kerry, T. Simpson, Large-scale bleaching of corals on the Great Barrier Reef. *Ecology* **99**, 501–501 (2018).

89. M. A. Chamberlain, P. R. Oke, R. A. S. Fiedler, H. M. Beggs, G. B. Brassington, P. Divakaran, Next generation of Bluelink ocean reanalysis with multiscale data assimilation: BRAN2020. *Earth Syst. Sci. Data* **13**, 5663–5688 (2021).
